# Comparative Study of Single-Cell and Bulk RNA Sequencing Data from Metastatic Bone Marrow Neuroblastoma Samples

**DOI:** 10.3390/cells15131139

**Published:** 2026-06-23

**Authors:** Sanja Aveic, Alessandro Davini, Sara Menegazzo, Marcella Pantile, Carlo Zanon, Anna Corrà, Giovanni Faggin, Diana Corallo, Danilo Pellin, Luisa Santoro, Chiara Frasson, Angelica Zin, Samuela Francescato, Bartolomeo Rossi, Ioana Ancuta Neculaescu, Martina Pigazzi, Barbara Buldini, Elisabetta Viscardi, Alessandra Biffi

**Affiliations:** 1 Pediatric Hematology, Oncology and Hematopoietic Cell & Gene Therapy Research Area, Pediatric Research Institute, Città della Speranza Foundation ETS, 35127 Padua, Italy; 2 Department of Women and Child’s Health, University of Padua, 35128 Padua, Italy; 3 Bioinformatics Core Service, Pediatric Research Institute, Città della Speranza Foundation ETS, 35127 Padua, Italy; 4 Gene Therapy Program, Dana-Farber/Boston Children’s Cancer and Blood Disorders Center, Boston, MA 02215, USA; 5 Anatomia Patologica, Azienda Ospedaliera Padova, 35121 Padua, Italy; 6 Flow Cytometry Core Service, Pediatric Research Institute, Città della Speranza Foundation ETS, 35127 Padua, Italy

**Keywords:** single cell, bone marrow metastasis, tumor antigens, neuroblastoma

## Abstract

Neuroblastoma is characterized by frequent involvement of bone marrow (BM) as a site of cell dissemination and spread. In this study, single-cell RNA sequencing (scRNA-seq) was used to analyze the cellular heterogeneity of a subset of metastatic BM samples collected at initial diagnosis. Comparison of the single-cell data with bulk RNA sequencing further refined the analysis. An enrichment of regulatory T cells relative to a healthy control and activation of the CD24, CD47, and CD200 “don’t eat me” signals were documented. Computational analyses highlighted communication between neuroblastoma and myeloid cells via the amyloid precursor protein (APP) and midkine (MK) signaling networks. Within neuroblastoma cells, mutually exclusive adrenergic and transitory cell states were identified, and ten sub-clusters were denoted. In addition, common and unique tumor cell antigens were investigated. CNTFR and CHRNA3, as high-ranking candidates, were validated, confirming their strong selectivity for neuroblastoma cells. Taken together, these findings support the existence of a significant tumor-dependent modulation of the BM ecosystem, which should be considered when introducing immunotherapy. Furthermore, they highlight the potential to investigate new antigens at the single-cell resolution.

## 1. Introduction

Neuroblastoma is the most common extracranial solid tumor in childhood and the most lethal malignancy in toddlers [1]. Neuroblastoma cells closely resemble embryonic progenitor cells known as neural crest cells (NCCs), and most likely originate from trunk NCCs positioned along the dorsal aorta, which differentiate into sympathetic neurons and neuroendocrine chromaffin cells of the adrenal medulla [2]. NCCs also contribute to the formation of various tissues, including cartilage, bone, muscle, connective tissue, the enteric nervous system, dorsal root ganglia, adrenal glands, and melanocytes [3]. Neuroblastoma is characterized by extreme heterogeneity in clinical presentation and biological background and a low mutational burden at disease onset [4,5]. Single-cell RNA sequencing (scRNA-seq) analyses of primary tumor masses have confirmed these findings, providing critical insights into the phenotypic diversity of tumor cells across primary and post-treatment samples [6,7]. Recent transcriptomic studies suggest that neuroblastoma genesis may occur before NCCs differentiate into sympathoadrenal cells [8], linking tumorigenesis with arrested fetal NCC development in pediatric cancers [9]. Furthermore, these studies have explored the neuroblastoma immunological landscape at the cellular level [10,11].

Over the past decade, novel treatment strategies, such as ALK kinase inhibitors and monoclonal antibody-based immunotherapy, have significantly improved the survival and quality of life of patients with high-risk neuroblastoma [12,13]. In addition, new clinical studies have provided promising indications of a possible breakthrough in the treatment of these patients by introducing stem cell transplantation or chimeric antigen receptor gene-modified T (CAR-T) cells targeting GD2 [14,15,16]. Furthermore, systemic characterization of primary tumors with a single-cell resolution has been achieved [7,8], opening new prospects for developing innovative therapeutic protocols against an updated list of molecular targets. All the progress made recently indicates potentially new hope for refractory and relapsed diseases, which continue to be largely fatal [17,18,19]. A major contributor to a poor prognosis in neuroblastoma patients is the high metastatic potential of the disease. Over 50% of patients present as metastatic at diagnosis, with bone and bone marrow (BM) serving as primary sites for malignant cell dissemination [20,21].

Metastatic neuroblastomas of the BM are generally less studied than primary tumors. However, the composition of the BM ecosystem is often modulated by tumor cells that promote the establishment of an immunosuppressive microenvironment [22]. In particular, the accumulation of myeloid-derived suppressor cells (MDSCs) has been linked to a poor prognosis for patients [23]. Meanwhile, tumor-associated neutrophils, regulatory T cells (Tregs), and exhausted T cells have recently been reported to suppress the anti-tumor immune response in the metastatic BM niche [22].

We performed single-cell profiling of infiltrated (naïve) BM samples to gain further insight into the composition and characteristics of neuroblastoma in high-risk metastatic patients [24]. We identified distinct tumor and non-malignant cell populations, as well as targetable signaling pathways instrumental to tumor survival and spread; explored the preferred interaction roadmaps between non-malignant and neuroblastoma cells in the BM; dissected the heterogeneity of tumor cell populations; and inspected the surface antigen diversity to identify potential new therapeutic targets.

## 2. Materials and Methods

### 2.1. Sample Collection and Patients’ Clinical Data

Cryopreserved bone marrow (BM) aspirates from four high-risk neuroblastoma patients and one pediatric healthy donor were utilized for single-cell RNA sequencing (scRNA-seq) using the BD Rhapsody™ platform. The viability cutoff was set at ≥65% post-thawing and ≥50% before cartridge loading to ensure sample integrity. Samples were obtained from treatment-naïve individuals, and the healthy donor sample was provided by the Biological Pediatric Oncology Biobank (BBOP) at the University Hospital of Padova. All procedures were ethically approved (approval number: 5289/AO/22). Written informed consent was obtained from parents or legal representatives, in accordance with the Declaration of Helsinki (2013 revision). Lack of written consent was an exclusion criterion for study enrollment. Total BM was analyzed without prior enrichment or depletion steps, ensuring an unbiased representation of the tumor microenvironment. The mean age at diagnosis for neuroblastoma patients was 37 months. MYCN amplification was detected in 50% of cases, consistent with its role as a high-risk neuroblastoma. All patients were classified as high-risk, based on INSS staging and molecular characteristics, except for one patient diagnosed with neuroblastoma MS. The clinical characteristics of the patients obtained through MFC and morphological evaluation are documented in Supplementary Table S1.

### 2.2. Cell Lines

The following human cell lines were used in this study: SH-SY5Y (DSMZ; Braunschweig, Germany), SK-N-BE(2) (ATCC; Manassas, VA, USA), SK-N-DZ (kindly provided by Prof. Elisa Cimetta, the University of Padua, Italy), IMR-32 (ATCC; Manassas, VA, USA), BJ-5ta (ATCC; Manassas, VA, USA), HS-5 (kindly provided by Prof. Katarzyna Piwocka, the Nencki Institute of Experimental Biology, Poland), and HeLa and MDA-MB-231 (both kindly provided by Dr. Elena Mariotto, the University of Padua, Italy). Detailed information on the media composition and culture conditions is provided in Table S2. All cell lines were routinely tested for mycoplasma contamination and authenticated using genotyping profiling at BMR Genomics, Padua, Italy.

### 2.3. Cryopreserved Primary Neuroblastoma Single-Cell Suspension Recovery

Cryopreserved primary neuroblastoma cells were thawed in a complete Neurobasal^®^ medium supplemented with 20% FBS (fetal bovine serum, Gibco, Milan, Italy), a protease inhibitor cocktail (cOmpleteTM, Mini, EDTA-free Protease inhibitor Cocktail, Roche, Monza, Italy), and an RNAse inhibitor (Roche, Monza, Italy). The cell suspension was dissociated with a cell strainer to eliminate cell clumps. Next, the cells were centrifuged at 1000 rpm for 5 min at 4 °C, and the pellet was washed twice with cold PBS supplemented with 0.5% BSA (Sigma-Aldrich, Milan, Italy). The cells were then resuspended in complete medium, and viability was measured using Trypan Blue (Invitrogen, Monza, Italy) exclusion labeling before proceeding with the BD Rhapsody and the MFC procedures.

### 2.4. Multiparametric Flow Cytometry (MFC)

Whole BMs were thawed and passed through a 40-micron strainer, and 0.5 × 10^6^ cells were prepared for MFC analysis. The cells were resuspended in 1× PBS with 5% bovine serum albumin (BSA, Sigma-Aldrich, Milan, Italy) for 30 min at 4 °C, according to internal standard operating procedures. The conjugated antibodies used for staining are listed in the antibody resource Table S3. The Tregs were denoted as CD3+/CD4+/CD25+ cells; NK cells (either CD56+ or CD16+) were denoted as CD7+/CD3−.

The samples were stained with 8- or 12-color antibody panels and detected with a FACS Canto II (BD Biosciences, Milan, Italy) or a FACSLyric (BD Biosciences, Milan, Italy) cytometer, respectively. At least 100,000 events/tubes were acquired. Analyses were performed using the Kaluza software (Beckman Coulter, Milan, Italy, v2.1). Immunophenotype was defined using a semi-quantitative method by comparing the fluorescence shift in the population of interest (neuroblastoma cells) with an appropriate internal control. Antigen expression was graded according to the WHO tripartite consensus rating (negative, weak, or strong) [25]. We used absolute frequencies and percentages for dichotomous and categorical variables. The BM cell subpopulations were considered only in hematopoietic compartments, excluding CD45−/CD56-high cells representing the neuroblastoma-invading population.

#### Flow Cytometry for TSA Validation

A total of 200,000 cells were detached with Accumax solution (Chemicon International, Millipore, Milan, Italy) and resuspended in 0.1% BSA/PBS to measure the surface antigen expression. The single-cell suspensions were stained with the primary antibodies for surface antigens for 20 min (anti-CHRNA 1:250; anti-CNTFRa 1:250). After 20 min of incubation, the cells were washed in PBS, resuspended in 0.1% BSA/PBS, and stained with the secondary antibody (Alexa-Fluor 488, Thermo Fisher Scientific, Milan, Italy 1:1000) for 20 min in the dark. The cells were analyzed with a CytoFLEX flow cytometer (Beckman Coulter, Brea, CA, USA), and the data were analyzed with the FlowJo software v7.6.5 (BD Bioscience, Franklin Lakes, NJ, USA).

### 2.5. Sample Processing, RNA Library Construction, and Single-Cell RNA Sequencing (scRNA-Seq)

For all samples, we assessed >50% viability necessary to proceed with single-cell capture on the BD Rhapsody™ platform [26]. Cell viability was tested with trypan blue immediately after thawing and with calcein-AM (Thermo Fisher Scientific, Milan, Italy) and Draq7 (BD Biosciences, Milan, Italy) before cartridge load. cDNA synthesis was assessed according to the Single-Cell Capture and cDNA Synthesis protocol recommended for BD Rhapsody Single-Cell Analysis (BD Biosciences, Milan, Italy). Half a million cells were centrifuged and resuspended in cold sample Buffer in accordance with the BD protocol for single-cell suspension preparation (BD Biosciences, Milan, Italy). The cells were co-labeled following the BD™ Single-Cell Multiplexing Kit protocol (BD Biosciences, Milan, Italy). After this step, the single-cell suspension was loaded into the BD Rhapsody™ cartridge. Single cells were captured with the BD Rhapsody™ Single-Cell Analysis System according to the BD Rhapsody™ scanner protocol (BD Biosciences, Milan, Italy), using at least 40,000 viable cells. Library construction was performed according to the prescriptions of the BD Rhapsody™ System mRNA Whole-Transcriptome Analysis (WTA) and Sample Tag Library Preparation (BD Biosciences, Milan, Italy). The library sequencing for the samples that passed quality control (QC) parameters was performed using Novaseq 6000 (Illumina, San Diego, CA, USA) on a 150 bp paired-end run in an outsource (Biodiversa, Padua, Italy).

### 2.6. scRNA-Seq Data Processing

The preprocessing of single-cell RNA-Seq data, which included cell barcode identification and UMI detection, extraction, and processing, was performed using UMI-tools 1.0.1 [27]. Read quality control (QC) analyses and filtering of high-quality reads were executed using FastQC (https://github.com/s-andrews/FastQC, accessed on 22 June 2022) and the Trimmomatic software (http://www.usadellab.org/cms/?page=trimmomatic, accessed on 22 June 2022), with the default options of a minimum length of 35 bp and a minimum quality score of 25 to perform adapter and quality trimming. STAR 2.7.3a was used to align the filtered reads to a human genome reference (GRCh38 Ensembl Release 105), while the FeatureCounts 1.6.3 package was applied to assign reads to genes.

Downstream analyses were performed according to Seurat (v4.3.0.1) protocols. For each sample, Seurat objects were created from raw count matrices and then merged. Features with fewer than 4 overall counts and cells showing mitochondrial RNA percentages of ≥30 and ribosome RNA percentages of ≤5 were discarded. After normalization, cell cycle scoring, SCTransformation, and PCA, the scRNA datasets were merged into a single Seurat object, and samples were integrated using Harmony v1.0.3. Dimensional reductions were calculated using the ‘RunUMAP’ function of Seurat with 3 components.

### 2.7. Annotation of Cell Populations in BM Using scRNA-Seq

Cell-type assignment was based on unbiased automatic annotation for each cell employing SingleR v2.2.0 [28], using data from the “nbl_target_2018_pub” TARGET program (https://www.cancer.gov/ccg/research/genome-sequencing/target/studied-cancers/neuroblastoma, accessed on 15 December 2022) as a reference dataset for neuroblastoma cells and from the GSE24759 dataset [29], retrieved via the ‘NovershternHematopoieticData’ SingleR function, as a reference for normal cells. Cells belonging to population assignments with fewer than 50 members were not considered in downstream analyses. The automatic annotations based on SingleR scores were further refined using a supervised approach for both normal and neuroblastoma cells. Normal cells annotated as ‘CMP’, ‘MEP’, and ‘HSC’ were redefined as ‘SC’. Neuroblastoma cells’ annotation was combined with a copy number variation (CNV) score of >0.2 and a neuroblastoma gene signature score of >0 (defined by PHOX2A, PHOX2B, TH, DBH, CHGA, CHGB, B4GALNT1, NCAM1, ISL1, PRPH, NPY, TCF21, RSPO3, WT1, INSM1, FOXD3, ERBB3, CARTPT, and HAND1 genes). Eventually, only ‘bona fide neuroblastoma cells’ were considered to meet all three criteria, discarding the rest as ‘undetermined’. The CNV profiles were inferred using the ‘infercnv’ v1.16.0 R package, and a CNV score was calculated as the mean across all chromosomes of their ‘proportion_cnv_chr’. Gene signature scores were calculated using the ‘AddModuleScore’ function of the Seurat package (v4.3.0.1).

### 2.8. Discovery of scRNA-Seq-Driven Tumor Cell-Associated Antigens

We devised a twofold strategy to identify differentially expressed genes (DEGs) between neuroblastoma and normal cells to identify potential tumor-associated surface antigens (TSAs). First, we compared all neuroblastoma cells against all normal cells from the BM as single groups to identify DEGs common to all neuroblastoma cells. Next, we compared each neuroblastoma sub-cluster with all normal BM cells to find DEGs specific to one/few neuroblastoma cell sub-clusters. All comparisons were carried out using the FindMarkers function of Seurat. After combining the DEGs resulting from the two approaches, we filtered them for the genes annotated as part of the Mass Spectrometric-Derived Cell-Surface Protein Atlas (CSPA database; https://wlab.ethz.ch/CSPA/, accessed on 18 June 2026) [30].

### 2.9. Pathway Analysis

Gene set enrichment analysis was performed with ‘enrichR’ v3.2 using the following reference databases: WikiPathway v.2023_Human, Reactome v.2022, KEGG v.2021_Human, GO Biological Process v.2023, and GO Molecular Function v.2023.

### 2.10. Receptor–Ligand Interactive Network Reconstruction

Receptor–ligand interactions were inferred and analyzed with the CellChat v1.6.1 R package using the human CellChatDB [31]. Interactions involving all cell populations in the NB metastatic BM were inferred and ranked by their ligand–receptor (L–R) probability. The top 10% significant (*p*-value ≤ 0.05) ones were selected and further ranked by their net contribution to the involved pathways. Only interactions involving ligands from tumor cells and the cognate receptors in the remaining non-tumoral cell populations were considered for downstream analyses. These investigations focused on the top interactions (with contribution values above the 75th percentile) and other L–R pairs (with probabilities above the 25th percentile) from the corresponding pathways.

### 2.11. Bulk RNA-Seq-Driven TSA Discovery

An in silico analysis was performed using a bulk RNA-seq dataset available at the Gene Expression Omnibus data repository (GEO ID: GSE94035) to validate the previously identified TSAs. This dataset contains data from a cohort of 54 patients affected by neuroblastoma classified as stage 4/M [32]. From these 54 patients, MNCs (BM-derived mononuclear cells), DTCs (BM-derived disseminated tumor cells), and TUs (primary tumor cells) were obtained, resulting in a total of 86 analyzed samples. The following comparisons were considered to identify DEGs: DTC vs. MNC and DTC&TU vs. MNC. DEGs between the two groups were identified via linear model analysis (‘limma’ package v 3.52.4). Only genes that met the criteria of adjusted *p*-value < 0.001 and │log2FC│ > 2 were selected as DEGs. Unsupervised clustering was performed to confirm the separation of the samples analyzed. An unsupervised heatmap and 3D PCA were used for data presentation. All the analyses and computational statistics were performed using the R programming language.

### 2.12. Immunofluorescence and Immunohistochemistry

An antigen-recognizing antibody was used to specifically label TSAs on the plasma membranes of the analyzed cells [33]. Briefly, the cell lines were detached with trypsin (PAN Biotech, Aidenbach, Germany), 5′ at 37 °C before seeding in P96-well plates with 100 µL of medium. Immunostaining was performed after 24 h. Neuroblastoma cells were fixed in 4% PFA for 15 min at room temperature. Slices were permeabilized with 0.3% Triton X-100 (Sigma Aldrich, Milan, Italy) for 15 min at room temperature and blocked in 5% BSA for 1 h at room temperature. Non-neuroblastoma cells were additionally labeled with a membrane-specific dye (MemBrite™ Fix Cell Surface Staining Kit, 30094-T, Biotium, Fremont, CA, USA) before fixation to confirm cell boundaries in case of negative signaling after antibody incubation. The cells were incubated with primary antibodies diluted in 1% BSA overnight at 4 °C, washed three times, and then incubated with secondary antibodies diluted in 1% BSA for 1 h at room temperature. The primary and secondary antibodies are listed in Table S3. Cell nuclei were stained with DAPI (Invitrogen, Milan, Italy). Slices were analyzed via confocal microscopy (Zeiss LSM 800 confocal microscope, Oberkochen, Germany). Images were processed using the IMAGE J software v1.54p (National Institutes of Health, Bethesda, MD, USA). Immunohistochemistry of 8 trephine biopsies from patients with stage M neuroblastoma at diagnosis was performed using double immunostaining for CD3 (murine monoclonal antibody, clone LN10, Novocastra, Leica Biosystems, Nussloch, Germany) and FOXP3 (ready-to-use primary antibody; Leica Biosystems, Nussloch, Germany). Hematoxylin and eosin staining was performed on formalin-fixed, paraffin-embedded tissue samples stained with hematoxylin–eosin.

### 2.13. Statistical Analysis

The Wilcoxon rank-sum test was used to determine the statistical significance of data with two levels. *p*-values of 0.05 or less were considered statistically significant. Statistical calculations were performed with R. *t*-test statistical analysis using the Prism software (v8.4.3) was used to analyze MFC data.

## 3. Results

### 3.1. Single-Cell Transcriptome Profiling of the Metastatic Neuroblastoma BM Niche

**Figure 1:**
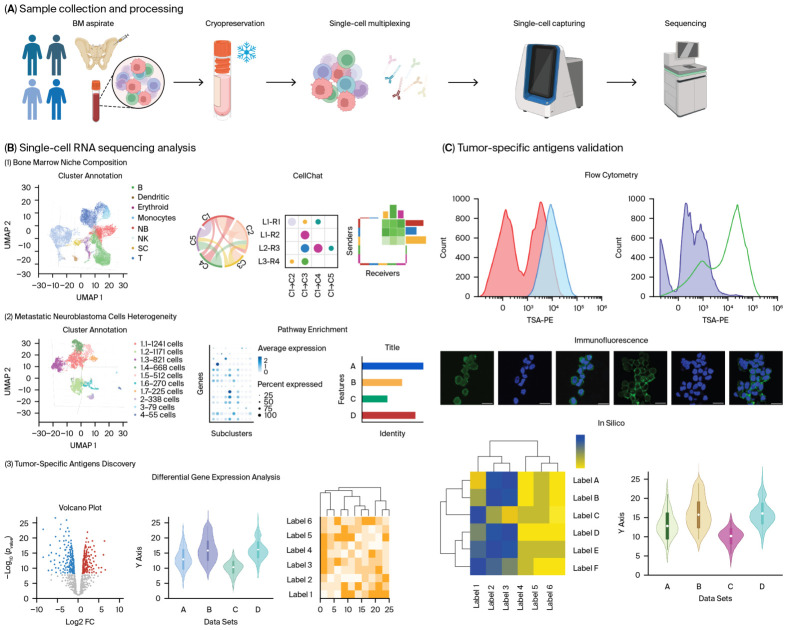
A schematic presentation of the experimental workflow starting from the (**A**) collection of BM specimens, sample processing, and cryobanking to single-cell RNA sequencing (scRNA-seq) and (**B**) data analysis and (**C**) validation.

The BM samples from four high-risk patients with neuroblastoma and metastatic disease at initial diagnosis (Table S1) and from a pediatric healthy subject were processed using the BD Rhapsody™ Single-Cell Analysis System for scRNA-seq (Figure 1A–C).

**Figure 2:**
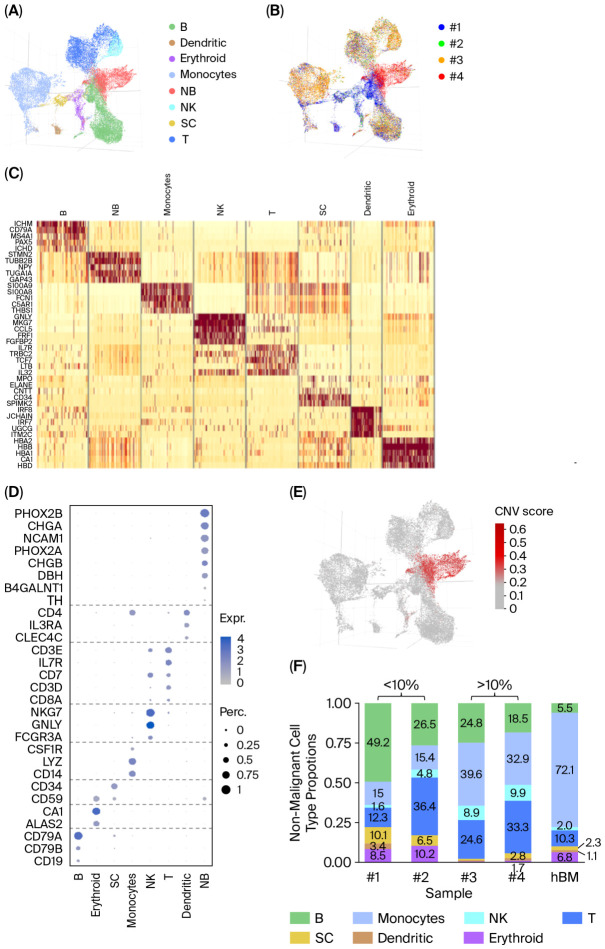
Investigation of neuroblastoma metastatic BM heterogeneity starting from single-cell RNA-seq data. (**A**) Three-dimensional UMAP visualization of the 42,531 single cells obtained from four BM metastatic specimens with distinct cell populations identified (color-coded). (**B**) Three-dimensional UMAP visualization of distinct cell populations obtained from four BM metastatic specimens (#1, #2, #3, and #4; color-coded). (**C**) Heatmap displaying the top 5 differentially expressed genes (DEGs) for each cell population identified in panel (**B**). Color code: Yellow—low expression, Red—high expression. Gene details are provided in Table S4. (**D**) Bubble plot showing the expression of the canonical genes across different cell populations from metastatic BM: CD79A, CD79B, and CD19 (B cells); CD3E, IL7R, CD7, CD8A, CD3D, and CD4 (T cells); FCGR3A, NKG7, and GNLY (NK cells); CD34 and CD59 (stem cells (SCs)); CD14, LYZ, CSF1R, and CD4 (monocytes); IL3RA, CLEC4C, and CD4 (dendritic cells); ALAS2 and CA1 (erythroid); and PHOX2B, PHOX2A, CHGA, CHGB, NCAM1, DBH, TH, and B4GALNT1 (neuroblastoma cells). The size of each dot indicates the percentage of expressed cells (Perc.), colored according to relative expression levels (Expr.). (**E**) Inferred CNVs based on single-cell RNA-seq data obtained by integrating the results from four patients. CNV score > 0.2 (red gradient) corresponds to the position of tumor cells in the 3D UMAP. (**F**) Bar plot comparing the proportions of non-malignant cells in four metastatic neuroblastoma BM samples with tumor cell infiltration <10% (samples #1 and #2) or >10% (samples #3 and #4), defined via MFC at the time of diagnosis versus healthy BM (hBM). Each cell population is color-coded, with percentages shown for major cell types (>0.5%).

The process included exclusive transcript counting via barcoding with unique molecular identifiers (UMIs) to distinguish different mRNA transcripts [34], followed by stringent quality control and doublet removal. The processed data were then projected using 3D Uniform Manifold Approximation and Projection (UMAP) plots. At the final stage, we obtained data from 12,940, 3745, 18,259, and 7586 cells for patients #1, #2, #3, and #4, respectively, collectively representing 36,128 unique transcripts. Patient-derived specimens were pooled using an unsupervised joint embedding-based integration to delineate the common cellular composition of the metastatic BM compared with control samples, yielding a final dataset of 42,531 cells for downstream analyses. Single-cell-level annotation was performed using SingleR [28]. Eight distinct cell populations were identified using computational methods (Figure 2A), including B cells (28.8%), dendritic cells (1.4%), erythroid cells (3.8%), monocytes (24.5%), natural killer (NK) cells (5.3%), hematopoietic stem/precursor cells (SCs; 4.2%), and T cells (19.4%). On average, 12.6% of tumor cells were also identified (ranging from 0.9% to 63.4%; Figure S1). The results were compared with the BM infiltration rates observed at the moment of diagnosis via morphological evaluation and multiparametric flow cytometry (MFC; Table S1 and Figure 2B).

The classification of the identified cell populations was further confirmed via an unsupervised analysis of the five top upregulated genes, which clustered each cell type (Figure 2C and Table S4). Moreover, all eight populations were ratified by analyzing the expression pattern of a short list of canonical genes (Figure 2D). Neuroblastoma tumor cells were additionally distinguished from non-malignant cell populations by estimating the inferred copy number variations (CNVs) based on transcriptome readouts (Figure 2E). This analysis revealed widespread copy number instability across the entire neuroblastoma cell population, confirming their malignant nature [35,36]. Computationally, a comparison of non-malignant populations in different BM samples showed cellular imbalances in multiple cell compartments (Figure 2F). A higher relative frequency of monocytes and NK cells was observed in samples with a rate of tumor infiltration at diagnosis superior to 10% (monocytes: 36.3 ± 4.7%; NK: 9.4 ± 0.7%) compared with those with less than 10% tumor infiltration (15.2 ± 0.3%; NK: 3.2 ± 2.2%; Figure 2F). Within monocytes, 22.4% of functional M1-like macrophages (CD68+, ITGAM+, CD80+, CD86+, FCGR1A+, FCGR2A+, FCGR2B+, and NOS2+) and 18.7% of M2-like macrophages (CD68+, ITGAM+, MRC1+, and CD163+) were estimated in silico in the metastatic BM samples. Meanwhile, they accounted for 2.7% (M1-like macrophages) and 0.9% (M2-like macrophages) in the healthy control (hBM). This result is consistent with recently published data [37]. Furthermore, the in silico data showed that all metastatic BM samples had lower monocyte and higher T and B lymphocyte frequencies than the healthy BM control (Figure 2F).

### 3.2. Neuroblastoma Cells Act as Signaling Senders in the Metastatic BM Niche

**Figure 3:**
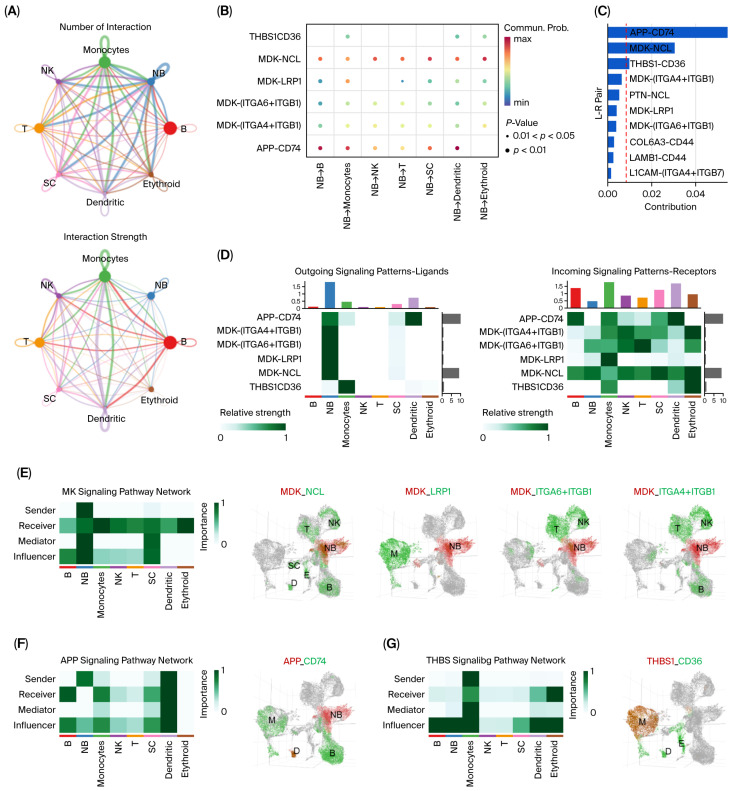
Cell–cell communication networks in metastatic neuroblastoma BM analyzed using CellChat. (**A**) Overview of intercellular interactions among the major cell populations in metastatic BM. The top circular diagram represents the number of interactions, while the bottom diagram illustrates interaction strength (probability). Larger circles indicate populations with greater involvement in intercellular communication. (**B**) Bubble heatmap displaying significant ligand–receptor (L–R) interactions between neuroblastoma (NB) cells and other BM populations. Dot size represents *p*-value significance, while the color gradient indicates communication probability. (**C**) Ranked list of the top ten L–R interactions across four metastatic BM samples. The red dashed line marks the 75th percentile, highlighting the most relevant interactions contributing to tumor microenvironment communication. (**D**) Heatmaps summarizing outgoing (left) and incoming (right) signaling patterns for different cell populations. The vertical axis lists L–R pairs, while the horizontal axis represents specific cell types that act as either signal senders (left panel) or receivers (right panel). The color intensity reflects the relative strength of communication across populations. (**E**–**G**) Detailed analysis of the three primary signaling pathways involved in tumor microenvironment interactions: (**E**) MK (midkine) signaling network: heatmap showing the relative importance of each cell type as a sender, receiver, mediator, or influencer. Adjacent UMAP plots display the spatial distribution of ligands (red) and receptors (green) within cell clusters. (**F**) APP (amyloid-beta precursor protein) signaling network: similar analysis highlighting the role of APP-CD74 interactions in immune cell communication. (**G**) THBS1 (thrombospondin-1) signaling network: heatmap and UMAP visualization demonstrating the involvement of THBS1-CD36 in ECM modulation and immune regulation. Abbreviations in 3D UMAP: M—monocytes; T—T cells; B—B cells; NK—natural killer; SC—stem cell precursors; D—dendritic cells; E—erythroid cells; NB—neuroblastoma cells.

We analyzed the expression levels of known ligand–receptor (L–R) pairs to infer preferential interaction pathways among the major cell populations and to investigate the cell–cell communication dynamics within the metastatic neuroblastoma BM niche [38]. Based on the number and strength of interactions among the eight most-represented cell populations, a wide range of global communication was found (Figure 3A). Functional association for each pair of interacting proteins (L–R) with ligands expressed by neuroblastoma cells, and the cognate receptor by any of the remaining BM non-malignant populations, was further considered in downstream analyses. Neuroblastoma cells established preferential communications with B cells, dendritic cells, and monocytes (dark red dots; Figure 3B) mostly through the amyloid-beta precursor protein (APP) and the midkine (MK) signaling pathways (Figure 3B,C). A third major communication network involved thrombospondin 1 (THBS1; Figure 3C), which is involved in extracellular matrix (ECM) remodeling and sustains a pro-inflammatory microenvironment [39]. Neuroblastoma cells primarily acted as signaling senders (Figure 3D, left), whereas monocytes were the main signal receivers (Figure 3D, right). Additionally, neuroblastoma cells exhibited an autocrine signaling loop via the MK pathway, suggesting that this signaling may contribute to their survival and proliferation. These findings were consistent across the analyzed patient samples (Figure S2A) and comparable to recent reports [37,40], indicating that the observed molecular interactions are relatively stable in the metastatic BM ecosystem.

### 3.3. The MK Signaling Pathway Is Favored by Neuroblastoma Cells

Focusing on the key ligand–receptor interactions in the metastatic BM samples, we identified computationally the following preferential signaling networks: (1) neuroblastoma cells predominantly engaged in interactions via MDK–NCL, MDK–LRP1, MDK–ITGA6 + ITGB1, and MDK–ITGA4 + ITGB1 (Figure 3E); (2) dendritic cells communicating with neuroblastoma cells via APP–CD74 (Figure 3F); and (3) monocytes interacting with neuroblastoma cells via THBS1–CD36, supporting a role in immune modulation and ECM remodeling (Figure 3G). These findings suggest that MK signaling serves as a central node in neuroblastoma-mediated tumor microenvironment interactions, potentially facilitating the reprogramming of neighboring immune and stromal cells. All the MK-related pathways were enriched in metastatic BM, further supporting their role in fostering a neuroblastoma-driven microenvironment [40,41,42,43,44]. The communication patterns in healthy BM significantly differed from those in metastatic BM. In particular, CXCL12–CXCR4 signaling was the predominant interaction in the healthy BM niche, whereas it was far less prominent in the metastatic samples (Figure S2B).

### 3.4. Immunosuppressive Surface Markers Are Enriched in Metastatic Neuroblastoma Cells

We employed multiparametric flow cytometry (MFC) on an expanded cohort of patients at diagnosis to further characterize the immunosuppressive signature of the metastatic BM samples (Table S1). This analysis identified common cell surface markers associated with cell-to-cell, cell-to-ECM, and immunomodulatory interactions (Table S5). The results revealed that neuroblastoma cells (CD45–/CD56-high gated population) in the metastatic BM niche robustly expressed pro-migratory and pro-adhesive receptors, including CD24, CD9, CD44, CD146, CD47, CD200, CD151, and CD90. This observation especially applied to CD24 (expressed by 9/9 specimens) and CD47 (expressed by 7/7 samples), both implicated in “don’t eat me” signaling, allowing tumor cells to evade immune clearance [45,46].

Significant expression of CD200 was also detected (expressed by 5/5 samples; Table S5), which, along with CD24 and CD47, most likely contributes to the establishment of an immunosuppressive tumor microenvironment in the metastatic BM niche [47,48].

### 3.5. Metastatic BM Is Enriched in Treg Cells

**Figure 4:**
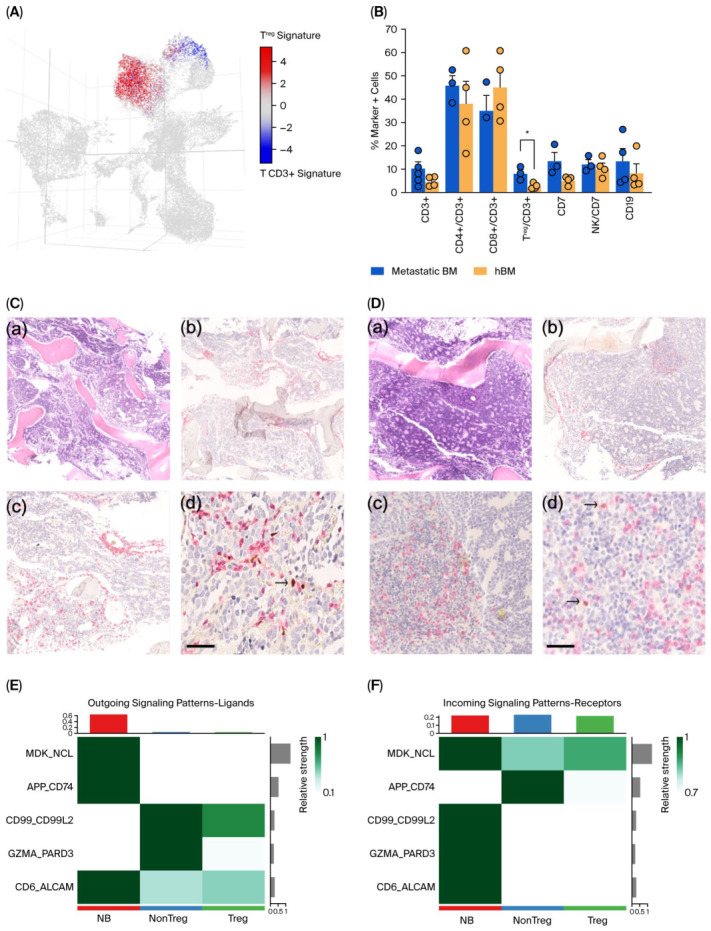
Treg cells in metastatic neuroblastoma. (**A**) A 3D UMAP of Treg (red color gradient) cells within the population of T cells (CD3+). (**B**) Multiparametric flow cytometry (MFC) data on BM cell populations in the BM with neuroblastoma invasion (in red; *n* = 4) and in healthy BM aspirates (hBM; in grey, *n* = 4) specimens per type (* *p* = 0.02). (**C**,**D**) Two representative cases of massive neuroblastoma infiltration of the BM with moderate (**C**) and mild (**D**) accumulation of T lymphocytes. ((**C**,**D**); (**a**)) Hematoxylin–eosin (magnification: 5×). ((**C**,**D**); (**b**,**c**)) Double immunostaining for CD3 (in red) and FOXP3 (in brown) (magnification: 5× and 10×). ((**C**,**D**); (**d**)) Higher magnification (20×) with isolated FOXP3-positive T lymphocytes (black arrows) (scale bar: 50 µm). (**E**) Outgoing and (**F**) incoming interaction strength between neuroblastoma (NB) and T cells (considered as either non-Treg or Treg subtype). The vertical axis indicates the ligand–receptor (L–R) pairs involved in signaling; the horizontal axis refers to the specific population that sends (upper panel) or receives (lower panel) the communication signaling. The histograms on the upper (0–0.6) and right (0–1) sides of the heatmap define the relative strengths of cell populations’ involvement as the sum of communication probabilities across L–R pairs (horizontal axis) and the relative strength of L–R pairs as the sum of communication probabilities across cell populations (vertical axis), respectively.

The in silico analysis also focused on T regulatory (Treg) cells, defined computationally as CD3+CD4+CD25+CD127+FOXP3+ within the T cell population of the metastatic BM niche (Figure 4A and Figure S3). The analysis revealed a five-fold increase in the frequency of Treg cells in the metastatic BM samples versus the healthy (control) BM (with mean values of 11.1% versus 2.3%, respectively). This increase in the relative frequency of Treg cells was confirmed via prospective characterization of the BM aspirates from the independent cohort of patients with metastatic neuroblastoma and healthy donors, analyzed via MFC (Figure 4B). In these samples, a significantly greater abundance of Treg cells was observed in metastatic relative to healthy BMs. Furthermore, a histological review of hematoxylin–eosin and immuno-stained BM trephine biopsies from six additional metastatic neuroblastoma samples confirmed this finding (Figure 4C,D). All cases showed mild-to-moderate accumulation of CD3+ T lymphocytes at the periphery and around the neoplasm. In all cases, a proportion of CD3+ T lymphocytes were also FOXP3+-positive throughout the metastatic BM tissue, as shown in the representative images of one case per pattern (moderate infiltration: Figure 4C(a–d); mild infiltration: Figure 4D(a–d)), supporting a recent report [49].

Based on this evidence, we continued to computationally analyze our single-cell data and focused on the communications between neuroblastoma cells and T lymphocytes, identifying differential pathways for Tregs and the other T cell subsets (non-Treg). While neuroblastoma cells showed preferential communication with the non-Treg subset via the APP–CD74 signaling pathway, they had preferential interactions with Treg cells via MDK–NCL signaling (Figure 4E,F), strengthening the role of this pathway in cell-to-cell communication in the metastatic BM niche [37].

### 3.6. Single-Cell Analysis Confirms the Heterogeneity of Neuroblastoma Cells Retrieved from the BM Metastatic Niche

**Figure 5:**
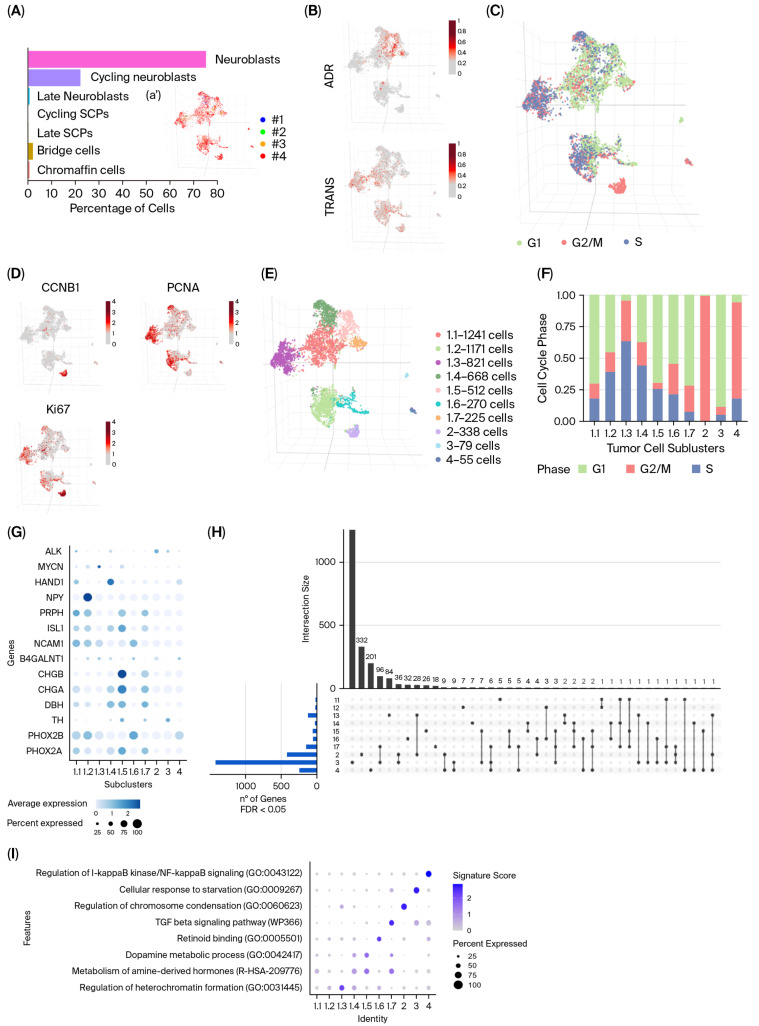
Characterization of the tumor cell population in metastatic neuroblastoma BM. (**A**) Proportions of neuroblastoma cells identified in metastatic BM, categorized based on their similarity to human fetal adrenal medulla-derived populations [7]. Subset (**a′**) represents neuroblastoma cells extracted from the BM and color-coded for each of the four patients (#1, #2, #3, and #4). SCP—Schwann cell precursors. (**B**) Three-dimensional UMAP visualization of adrenergic (ADR) and transitory (TRANS) signatures in metastatic neuroblastoma cells, as defined by Yuan et al. [6]. (**C**) Cell cycle scattering profile in neuroblastoma tumor cells showing individual cells assigned to G1, S, or G2/M phases. (**D**) Three-dimensional UMAP plots depicting the expression of key genes involved in cell cycle regulation (CCNB1) and proliferation (PCNA and MKI67). (**E**) Neuroblastoma cells organized into ten distinct sub-clusters using Seurat analysis. The number of cells within each sub-cluster is indicated. (**F**) Cell cycle phase distribution across the ten neuroblastoma sub-clusters, represented as bar plots. (**G**) Expression patterns of neuroblastoma-specific markers across tumor sub-clusters, shown as a dot plot where dot size indicates percentage of cells expressing each gene, and color intensity represents average expression level. (**H**) UpSet plot displaying marker gene distribution across tumor sub-clusters. Blue bars represent the number of marker genes identified per sub-cluster, while black bars indicate intersection sizes between different sub-clusters, with connected dots showing shared gene sets. The number of marker genes in each intersection is labeled above the respective bars. (**I**) Pathway enrichment analysis of activated signaling pathways in neuroblastoma sub-clusters, integrating Gene Ontology (GO), WikiPathways, and Reactome databases. Only pathways with an adjusted *p*-value of ≤0.05 are included. Dot size indicates percentage of expressing cells, and color intensity reflects signature score.

Neuroblastoma is characterized by high intra-tumoral heterogeneity, which significantly influences tumor progression and therapy resistance [50]. We used the single-cell transcriptomic data to classify tumor cells based on their transcriptional profiles to better understand the molecular diversity of malignant cells in BM metastases. Using de novo clustering, we found that most neuroblastoma cells in BM metastases were transcriptionally aligned with neuroblasts and cycling neuroblasts of the developing adrenal medulla (Figure 5A), as defined by Jansky et al. [7]. In contrast, only a small subset of tumor cells exhibited transcriptional signatures resembling bridge or chromaffin cells among the analyzed patients (Figure 5A and Figure S4). We further refined the phenotype of the metastatic neuroblastoma cells only (Figure 5(a′)) by challenging them with recently proposed signatures able to recognize mesenchymal, transitory (also known as mixed), and adrenergic neuroblastoma cells on the primary tumor material [6,51]. Our analysis revealed that metastatic BM neuroblastoma cells formed mutually exclusive clusters of adrenergic and transitory cells (Figure 5B). The mesenchymal signature was not detected in the malignant BM population; instead, it was confirmed in non-malignant stromal populations, in both the metastatic (Figure S5A) and healthy (Figure S5B) BM. These findings confirm that the established mesenchymal signature does not apply to neuroblastoma cells in BM metastases [37,52], contrary to observations in primary tumors [6].

Further evaluation of the expression levels of master cell-cycle regulatory genes showed that most tumor cells in the BM samples were in the G1 phase (45.1%), with only a minor fraction being in either the S (31.9%) or G2/M (23.0%) phases (Figure 5C). The expression of the cell-cycle regulatory gene CCNB1 (G2/M phase transition) and G1/S phase marker PCNA [53] was consistent with the expression of the proliferation marker MKI67 in S or G2/M cells (Figure 5D), suggesting that the fraction of proliferating tumor cells may promote uncontrolled cell growth. In our samples, non-proliferative (dormant; G0 phase) cancer stem cells characterized by the expression of PROM1, SOX2, and ALDHA1 genes could not be clearly identified/quantified.

Further scrutiny of the malignant cell population within the BM metastases identified ten distinct tumor cell sub-clusters, which were present in different percentages across the four specimens; sub-clusters 3 and 4 were found exclusively in sample #4 (Figure 5E and Figure S6). These sub-clusters exhibited different cell cycle states, with clusters 1.1, 1.5, 1.6, 1.7, and 3 predominantly in the G1 phase, clusters 1.2, 1.3, and 1.4 in the S phase, and clusters 2 and 4 in the G2/M phase (Figure 5F). In addition to cell cycle differences, we observed high heterogeneity in the expression of conventional neuroblastoma markers across sub-clusters (Figure 5G). Their distribution is further highlighted in Figure 5H, demonstrating that these tumor subpopulations are transcriptionally distinct. We performed a pathway enrichment analysis using Gene Ontology (GO), WikiPathways, and Reactome databases to explore the functional differences among the tumor sub-clusters. This analysis revealed that cluster 1.3 was enriched for heterochromatin formation (GO:0031445); cluster 1.6 showed significant activity in retinoid binding (GO:0005501); cluster 1.7 was enriched for the TGF-beta signaling pathway (WP366); cluster 2 exhibited enrichment in chromosome condensation regulation (GO:0060623); cluster 3 showed signatures related to cellular response to starvation (GO:0009267); and cluster 4 displayed activation of NF-kappaB signaling (GO:0043122) (Figure 5I). Tumor sub-clusters 1.1, 1.2, 1.4, and 1.5 displayed a combination of multiple GO pathway enrichments, highlighting their transcriptional plasticity (Figure 5I). The enrichment of NF-kappaB signaling in sub-cluster 4 suggests that this sub-population may be particularly relevant for cellular persistence in heavily infiltrated BM and that this signature may point to relevant biological processes that influence the phenotype, since NF-kappaB has been implicated in tumor survival, inflammation, and therapy resistance [54].

### 3.7. The BM Metastatic Neuroblastoma Cell Surface Signature Can Be Defined at the Single-Cell Resolution

**Figure 6:**
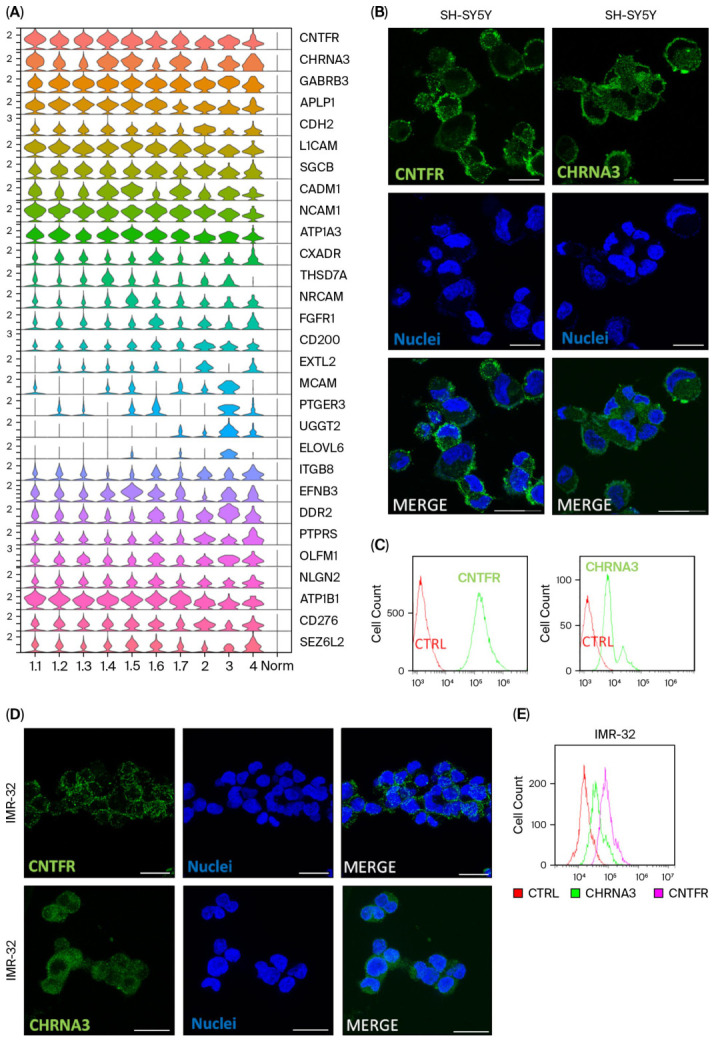
Prediction and validation of neuroblastoma TSAs. (**A**) A violin plot of the expression of 29 transcripts defining TSAs in each of the ten neuroblastoma cell sub-clusters (1.1; 1.2; 1.3; 1.4; 1.5; 1.6; 1.7; 2; 3; and 4). Norm—normal cell populations from the BM metastatic neuroblastomas. (**B**) Representative immunoreactivity and (**C**) flow cytometry readouts for CNTFR and CHRNA3 TSAs validated on the membrane of the non-MYCN-amplified neuroblastoma cell line SH-SY5Y. Immunofluorescence: green—488 coupled secondary antibodies. DAPI (blue)—nuclear staining. Scale bar: 20 μm. Flow cytometry: green signal refers to a specific TSA; red signal refers to control cells stained with secondary antibody only. (**D**) Representative images of immunofluorescence staining for MYCN-amplified neuroblastoma cell line IMR-32. Scale bar: 20 μm. (**E**) Representative flow cytometry readouts for the quantification of the CNTFR (green) and CHRNA3 (pink) TSAs versus control samples (red; incubated with secondary antibody only). Y-axis—cell number.

We performed a comprehensive single-cell transcriptomic analysis integrated with surface marker profiling to identify the cell surface antigenic landscape of neuroblastoma cells in BM metastases. We discovered tumor surface antigens (TSAs) that were either shared across all ten tumor sub-clusters or restricted to specific neuroblastoma sub-clusters (Figure 6A). These TSAs were exclusively expressed by malignant cells (Figure 6A). We focused on the top 29 TSAs that were significantly overexpressed in malignant versus non-malignant cells (Table S6). The TSAs expressed across all ten tumor sub-clusters included the following: the neuroblastoma-associated adhesion molecules and receptors CNTFR, CHRNA3, L1CAM, NCAM1, CADM1, EFNB3, FGFR1, NRCAM, and SEZ6L2; the ion transporters and synaptic proteins ATP1B1, ATP1A3, GABRB3, APLP1, and CXADR; the extracellular matrix and cell adhesion proteins SGCB, OLFM1, DDR2, CDH2, CD276, PTPRS, and CD200; and the signaling molecules and transporters NLGN2 and ITGB8. The TSAs restricted to specific tumor sub-clusters included THSD7A, PTGER3, MCAM, UGGT2, EXTL2, and ELOVL6. The expression of these TSAs was minimal or absent in non-malignant BM cell populations from the same patient cohort (norm; Figure 6A), supporting their tumor-specific nature. When analyzed for each patient individually, a heterogeneous distribution was observed for a single TSA (Figure S7).

We validated our findings at the protein level by selecting two less-characterized TSAs from the top-ranked TSAs, CNTFR and CHRNA3, both relevant to metastatic neuroblastoma [55,56]. Immunofluorescence staining and MFC were performed on a panel of neuroblastoma cell lines with distinct molecular backgrounds (Figure 6B–E and Figure S8A,B; MYCN-amplified cell lines: IMR-32, SK-N-BE(2), SK-N-DZ, and MYCN; non-amplified cell lines: SH-SY5Y and SK-N-AS). Both approaches confirmed robust CNTFRα expression and moderate CHRNA3 expression on the cell membranes of all neuroblastoma cell lines analyzed (Figure 6B,C and Figure S8A,B). We examined the expression of these TSAs in non-malignant stromal cells and other tumor cell types to assess their specificity. Stromal cells (BJ-5ta and HS-5) showed negligible or absent expression of CNTFR and CHRNA3 (Figure S9A,B). Other tumor cell types, including cervical cancer (HELA; Figure S10A) and breast cancer (MDA–MB–231; Figure S10B), exhibited minimal or no expression of the two TSAs.

### 3.8. Bulk RNA-Seq Data Summarizing the Results Obtained at the Single-Cell Level

**Figure 7:**
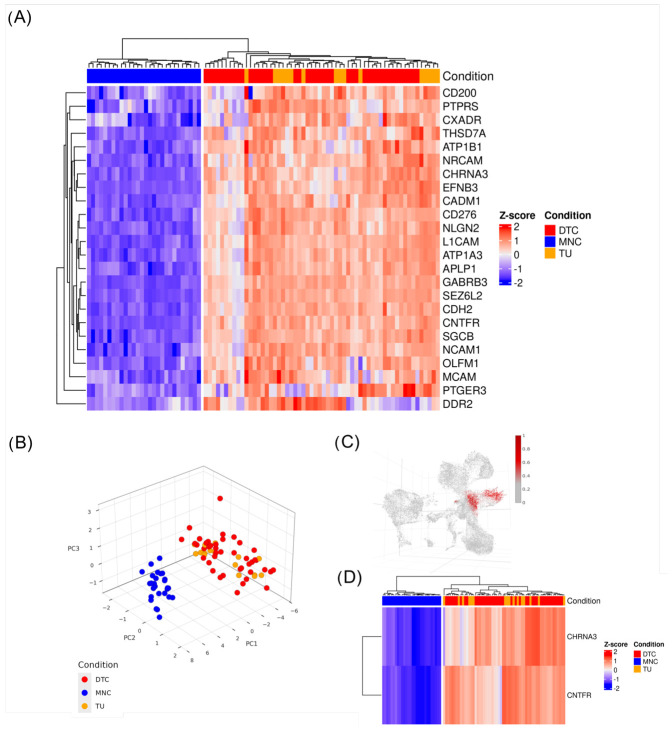
In silico validation of scRNA-seq-delineated TSA specificity in the bulk RNA-seq dataset (GSE94035). (**A**) Heatmap of bulk RNA-seq expression z-scores computed for the 24-TSA signature. Disseminated tumor cells—DTCs (red); primary tumor cells—TUs (orange); non-malignant mononuclear cells—MNCs (blue). Overexpression corresponds to │log2FC│> 2; *p*-value < 0.001. (**B**) Three-dimensional principal component analysis (PCA) of tumor-related (DTCs—red; TUs—orange) and non-malignant (MNCs—blue) cells from the metastatic BM based on the expression of 24 TSAs. (**C**) Validation of 24-TSA signature on scRNA-seq data. Color gradation: Z-score—from minimum (0; gray) to maximum (1; red). (**D**) Unsupervised clustering analysis (heatmap) showing the distribution of the two validated TSAs in a group of tumor samples (DTCs—red; TUs—orange) or non-malignant mononuclear cells (MNCs—blue). Upregulation is highlighted in red, and downregulation in blue.

We further validated the role of TSAs derived from a small set of samples by inferring their expression using bulk RNA-seq data (GEO ID: GSE94035), which included transcriptomic data from 86 specimens comprising primary tumor-derived cells (TUs), BM-derived disseminated tumor cells (DTCs), and BM-derived mononuclear cells (MNCs) [32]. The data matrix reported the expression levels of 45,113 transcripts for each of the 86 samples. We analyzed genes overexpressed in the DTC group (and eventually in the TU group) but not expressed in the MNC control group (Table S7) to search for the 29 TSAs selected from scRNA-seq data. The following comparisons were considered: DTCs versus MNCs and DTCs&TUs versus MNCs. Twenty-four of the differentially expressed genes (DEGs) found in bulk RNA-seq were also found in scRNA-seq data (Figure 7A), including the two validated TSA candidates, CHRNA3 and CNTFR. Computational analysis revealed average expression levels of CNTFR and CHRNA3 of 0.7 ± 0.3 in MNCs and 6.9 ± 0.5 and 7.0 ± 0.3 in DTC and TU, respectively. The 24 genes encoding potential TSAs could distinguish MNCs from DTCs&TUs, confirmed via unsupervised clustering analysis (heatmap; Figure 7A) and 3D principal component analysis (PCA; Figure 7B). Moreover, the 24 TSAs could specifically annotate neuroblastoma cells in single-cell data without interfering with the non-malignant cell populations from metastatic BM (Figure 7C). The signature could distinguish metastatic tumor cells at both levels: the total population of neuroblastoma cells in BM (Figure 7C) and singularly for each patient analyzed (Figure S11). When the 24 TSA signature was annotated on the healthy BM, only a small percentage of cells from different populations was positive (Figure S12), confirming a high specificity of the inferred TSAs. The expression of the two validated TSAs was weak in MNCs (Figure 7D), strengthening their potential as candidates for further exploration in immunotherapeutic targeting. The signature of the 24 TSAs predicted in BM metastatic neuroblastomas was then computationally explored in the cohort of primary tumors from publicly available datasets [57], confirming their limited expression in non-tumor cell populations (Figure S13A). When analyzed individually, these TSAs showed a heterogeneous distribution across three annotated tumor cell clusters from localized tumors (Figure S13B). Meanwhile, they were largely absent in normal cell populations (e.g., endothelial cells or leukocytes), except for DDR2, which also showed relevant expression in the mesenchyme.

## 4. Discussion

Despite being a rare tumor, neuroblastoma is characterized by great cellular heterogeneity and plasticity. The extent and causes of this diversity are not yet fully understood. This feature applies to both primary tumors and metastatic lesions, particularly those infiltrating the BM. We applied scRNA-seq to systematically decipher the heterogeneity of BM metastatic neuroblastoma, enabling the annotation of tumor cells and the complex microenvironment of non-malignant cell populations. Our findings revealed different relevant characteristics of the tumor and its microenvironment. Firstly, we confirmed the well-known tumor heterogeneity, identifying ten distinct tumor sub-clusters in our samples, which partially overlap in their transcriptional profiles and predominantly resemble neuroblasts and cycling neuroblasts, consistent with their sympathoadrenal developmental origin [7]. Although some sub-clusters shared features with cells of the developing human adrenal medulla, the presence of multiple transcriptionally distinct tumor cell sub-populations suggests that intra-tumor heterogeneity extends beyond the primary tumor and is maintained within the BM metastatic niche [4,7]. The presence of Schwann cell precursor (SCP)-like cells—a multipotent cell type derived from sympathoblasts during adrenal development [58]—was low in our dataset. Furthermore, we did not detect a mesenchymal gene expression signature, which is typically enriched in SCP-like populations in non-BM localized tumors. Instead, our data indicate that BM metastatic neuroblastoma comprises a mixture of proliferating neuroblasts and differentiating adrenergic and transitory tumor cells [8], maintaining phenotypic cell plasticity within the BM ecosystem, similar to solid tumor masses [57,59]. The existence of multiple tumor sub-clusters within BM metastases likely reflects a functional diversity that allows neuroblastoma cells to adapt to the BM microenvironment, potentially contributing to differential responses to therapy. Given the low mutational burden of neuroblastoma [60], intra-tumor heterogeneity at the transcriptional and epigenetic level may serve as an alternative mechanism driving tumor progression and therapy resistance. However, other factors, including immune diversity and stromal interactions, may influence this diversity and be critical determinants of patients’ prognosis and treatment response.

Next, we identified the key source of communication in the niche and a small subset of preferential ligand–receptor (L–R) interactions in neuroblastoma cells by systematically mapping tumor and immune cell interactions in the BM microenvironment. Specifically, amyloid-beta precursor protein (APP) and midkine (MK) signaling pathways emerged as dominant mediators of neuroblastoma outgoing communications, agreeing with recent evidence implicating neuroblastoma cells as key modulators of BM microenvironmental signaling [37]. Despite the intra-tumor heterogeneity observed in our dataset, the preferential communication networks between tumor and non-malignant BM populations were conserved across patients, suggesting that these interactions play a functionally significant role in sustaining tumor growth and immune evasion. Therapeutically, these signaling pathways represent potential targets for enhancing the sensitivity of BM metastatic neuroblastoma cells to systemic and immunotherapies and could be explored further. For example, the targeted inhibition of CD74 within the APP-CD74 axis has been proposed as a potential therapeutic approach for glioblastoma multiforme and B-cell non-Hodgkin lymphoma [61,62]. Based on our results and those of other research groups [37], this axis represents a plausible new vulnerability in neuroblastoma that should be considered for therapeutic interventions. By examining the non-tumor components of the niche, we observed an enrichment of myeloid-derived populations in BM samples with higher tumor infiltration. Pro-inflammatory M1-like and anti-inflammatory M2-like phenotypes were distinguished among the macrophages by analyzing the transcriptional profile of these cells. The M2 phenotype was more pronounced in the metastatic BM than in the healthy counterpart, suggesting that neuroblastoma cells may actively shape the immune landscape by promoting the recruitment or expansion of immunosuppressive myeloid cells. Previous studies have reported that BM metastatic neuroblastoma is infiltrated by T, B, and NK cells, yet is also enriched in suppressive myeloid populations that counteract immune-mediated tumor clearance [63]. In addition to M2-type macrophages, we documented the preferential presence in the metastatic niche versus a healthy counterpart in T regulatory (Treg) cells, confirming recent findings [22]. This result was consistent across in silico and prospectively generated data and was also confirmed on pathological specimens such as trephine biopsies. The confirmation of Treg cell abundance in our samples by MFC could significantly contribute to the debate on Treg cells’ role in neuroblastoma, as the information on the presence and activity of true Treg cells in the primary and metastatic tumor, and the evidence of their immunosuppressive function in the tumor microenvironment, is scarce and influenced by the cell identification method [64]. However, it remains to be determined whether the pre-existing BM microenvironment (i.e., high or low in B, NK, or other cells) influences the transition between active (present in “hot” tumors) and reduced (present in “cold” tumors) immunogenicity [65] and how Tregs contribute to the transition towards suppression of immune activity in neuroblastoma.

These findings underscore the complexity of the metastatic niche and its prevalent immunosuppressive features sustained by multiple signaling pathways, mostly originating from tumor cells. They also support the importance of a detailed characterization of the tumor microenvironment in high-risk neuroblastoma patients, as immune cell composition may inform personalized targeted therapeutic strategies [63,66,67]. Moreover, given that neuroblastoma and other solid malignancies exhibit an immunosuppressive microenvironment that contributes to immunotherapy resistance [68], the identification of several immunosuppressive signals (such as CD24, CD47, and CD200) within neuroblastoma metastases in the BM highlights their plausible role in modulating/limiting the efficacy of these approaches. Specifically, the CD24, CD47, and CD200 signals are likely involved in tumor immune evasion via MK signaling and “don’t eat me” pathways, enabling neuroblastoma cells to escape immune-mediated clearance [69]. These findings suggest that neuroblastoma cells reshape their microenvironment via ligand–receptor signaling. Furthermore, they may actively suppress immune responses via the expression of immune checkpoint molecules, thereby enhancing their survival in the BM niche. Therefore, targeting these molecules in combination with immunotherapies may enhance anti-tumor activity [70,71]. The targeted inhibition of CD200 using the monoclonal antibody OX90 shows promise for future immunotherapies, as it enables the immune system to attack malignant cells more effectively and reduce the number of Treg cells [72]. However, the inclusion of CD47 should be viewed with caution, given the results of magrolimab, a first-in-class humanized anti-CD47 monoclonal antibody of immunoglobulin G4 that has been tested in acute myeloid leukemia, which have raised safety concerns that are under evaluation [73].

These markers did not necessarily define cancer stem cells characterized by the expression of PROM1, SOX2, and ALDHA1 genes, which were not detected in silico in our samples. This may be due to a loss of these cells during the preparation of the bio-banked material or because these tumor stem cells may not reside in the BM. Our data support the role of Treg cells in the BM of patients with neuroblastoma, as their presence may correlate with an immunosuppressive ecosystem in the metastatic disease [49,74] and potentially impair immune cell function in solid tumors [75]. Thus, strategies aimed at modulating Treg activity in the BM niche may also be important for improving immunotherapeutic responses in high-risk neuroblastoma patients.

Our study also represents a significant effort using scRNA-seq for the systematic identification of TSAs in pediatric neuroblastoma, potentially impacting the development of novel immunotherapy applications [76]. Our single-cell analysis provided a list of bona fide TSAs, several of which were previously linked to neuroblastoma progression, including the two validated antigens CNTFR and CHRNA3 [56,77,78,79,80]. The TSA list was cross-validated using bulk RNA sequencing and MFC in neuroblastoma cell lines and patient-derived BM samples, demonstrating strong concordance across different methodologies, as also shown in other similar reports [81]. This approach supports the reliability of single-cell transcriptomics for biomarker discovery, despite the inherent limitations of the sample size. Our TSA list correctly distinguished tumor cells from other non-tumor cell populations, suggesting that the proposed TSAs could contain promising candidates for identifying neuroblastoma cells disseminated from the primary tumor into the BM and, eventually, their targeting [82]. Among these top-ranked TSAs, B7-H3 (CD276) has garnered interest as a highly promising CAR-T therapy target, showing potent anti-cancer activity in preclinical models [83,84]. It is currently being tested in an early-phase clinical investigation (ClinicalTrials.gov: NCT04897321) [85]. Further preclinical studies are required to validate the protein-level abundance of the identified TSAs in larger patient cohorts, the off-target toxicity risks associated with individual TSAs, and the functional impact of TSA blockade on tumor cell survival and immune evasion. However, the integrative approach we adopted in our study may accelerate immunotherapy development in neuroblastoma. This may be relevant for the combinatorial approach using a CAR-T cell with two specificities. One such approach is a GD2-gated CAR-T targeting B7-H3, developed as an alternative treatment for metastatic neuroblastoma, performing better than conventional CAR-T [86]. A similar method may be considered for other antigens relevant to neuroblastoma, such as GPC2, L1CAM, and ALK [87,88,89].

## 5. Conclusions

Overall, our study contributes to an improved understanding of cellular heterogeneity, tumor–immune interactions, and immunosuppressive mechanisms in BM metastatic neuroblastoma that could inform future targeted therapies. By enlisting numerous TSAs and validating their expression in several cell types, we pave the way for expanded immunotherapy applications and support the development of dual-targeting strategies to overcome the observed immune escape mechanisms. Moving forward, integrating functional validation studies with immune profiling will be essential for translating these findings into clinically actionable immunotherapies.

## Data Availability

The data are available in the public, open-access repositories. All raw scRNA-seq data are deposited in NCBI SRA (accession code: PRJNA1327638). The processed external dataset (accession numbers: GSE24759 and GSE94035) and processed data were obtained from http://neuroblastomacellatlas.org (accession codes: PRJEB41516 and ERP125307, accessed on 15 December 2022). This study did not report novel or original codes.

## Supplementary Materials

The following supporting information can be downloaded at: https://www.mdpi.com/article/10.3390/cells15131139/s1, Figure S1: Major cells’ type distribution; Figure S2: Inferred cell-cell communication by CellChat; Figure S3: Treg signature genes expression; Figure S4: Proportions of neuroblastoma cells in metastatic BM; Figure S5: Mesenchymal (MES) signature distribution in the cell populations from; Figure S6: Proportions of neuroblastoma cells in metastatic BM; Figure S7: Expression patterns of TSA across analyzed specimens as dot plot; Figure S8: Expression of TSAs in different neuroblastoma cell lines; Figure S9: Expression of TSAs in non-malignant cells; Figure S10: Expression of TSAs in non-neuroblastoma cancer cells; Figure S11: Assessment of 24 TSAs signature in the individual patient’s BM specimens (numbers #1, #2, #3 and #4 indicate sample origin and correspond to the same samples described in Figure 2F); Figure S12: Assessment of the 24 TSAs signature (TSA score) in the principal cell macro populations of healthy BM specimen (normal) and patient derived samples (pts); Figure S13: Assessment of 24 TSA signature A) as a signature (all together) or B) individually, in the publicly available scRNA-seq dataset obtained from diagnostic biopsies or neuroblastoma resections of primary tumors; Table S1: Clinical features of patients; Table S2: Cell lines and media; Table S3: List of the antibodies used for immunostaining and MFC; Table S4: Seurat ‘FindAllMarkers’; Table S5: Immunophenotypic profile based on MFC for neuroblastoma cells infiltrating BM; Table S6: ‘FindAllMarkers’ output of the TSA genes; Table S7: Differentially expressed genes (DEGs) detected in bulk RNA-seq.

## Abbreviations

The following abbreviations are used in this manuscript:

TSA	Tumor surface antigens
APP	Amyloid precursor protein
MK	Midkine pathway
scRNA-seq	Single-cell RNA sequencing
MDSCs	Myeloid-derived suppressor cells
Treg	Regulatory T cells
UMIs	Unique molecular identifiers
MFC	Multiparametric flow cytometry
