# Supplementary material for: Comparative Study of Single-Cell and Bulk RNA Sequencing Data from Metastatic Bone Marrow Neuroblastoma Samples
Source: Cells. 2026 Jun 23;15(13):1139. doi: 10.3390/cells15131139 (PMC13359927; doi:10.3390/cells15131139)
Supplement: Supplementary file 1 [file cells-15-01139-s001.zip › Aveic et al_supplementary__R1_final_20Jun2026.pdf]

Supporting Information

Supplementary Figures

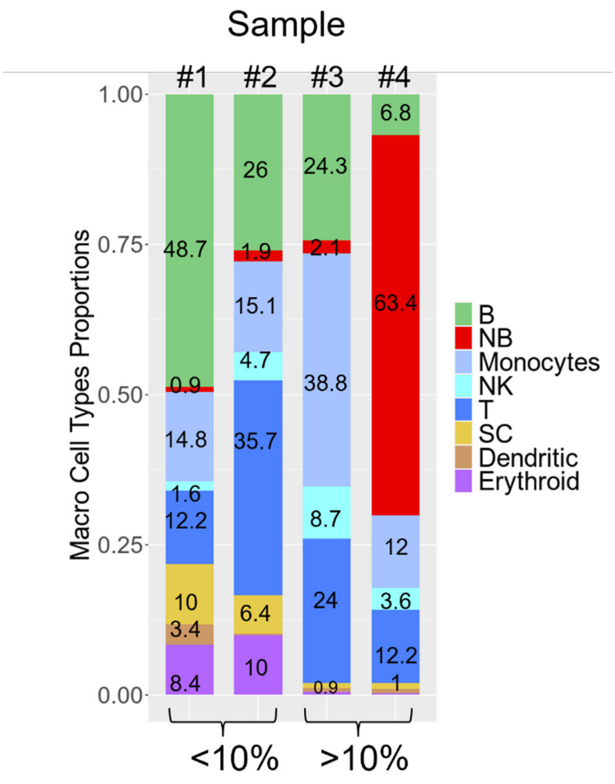

Figure S1. Major cells' type distribution. A bar plot comparing the proportions of neuroblastoma and non-malignant cells of four metastatic neuroblastoma BM samples with infiltration of tumor cells <10% (samples #1 and #2) or >10% (samples #3 and #4), defined by MFC at the time of diagnosis. Each cell population is color-coded, with percentages shown for major cell types (percentage above 0.5%). Abbreviations: T – T cells; B – B cells; NK – natural killer; SC – stem cell precursors; NB – neuroblastoma cells.

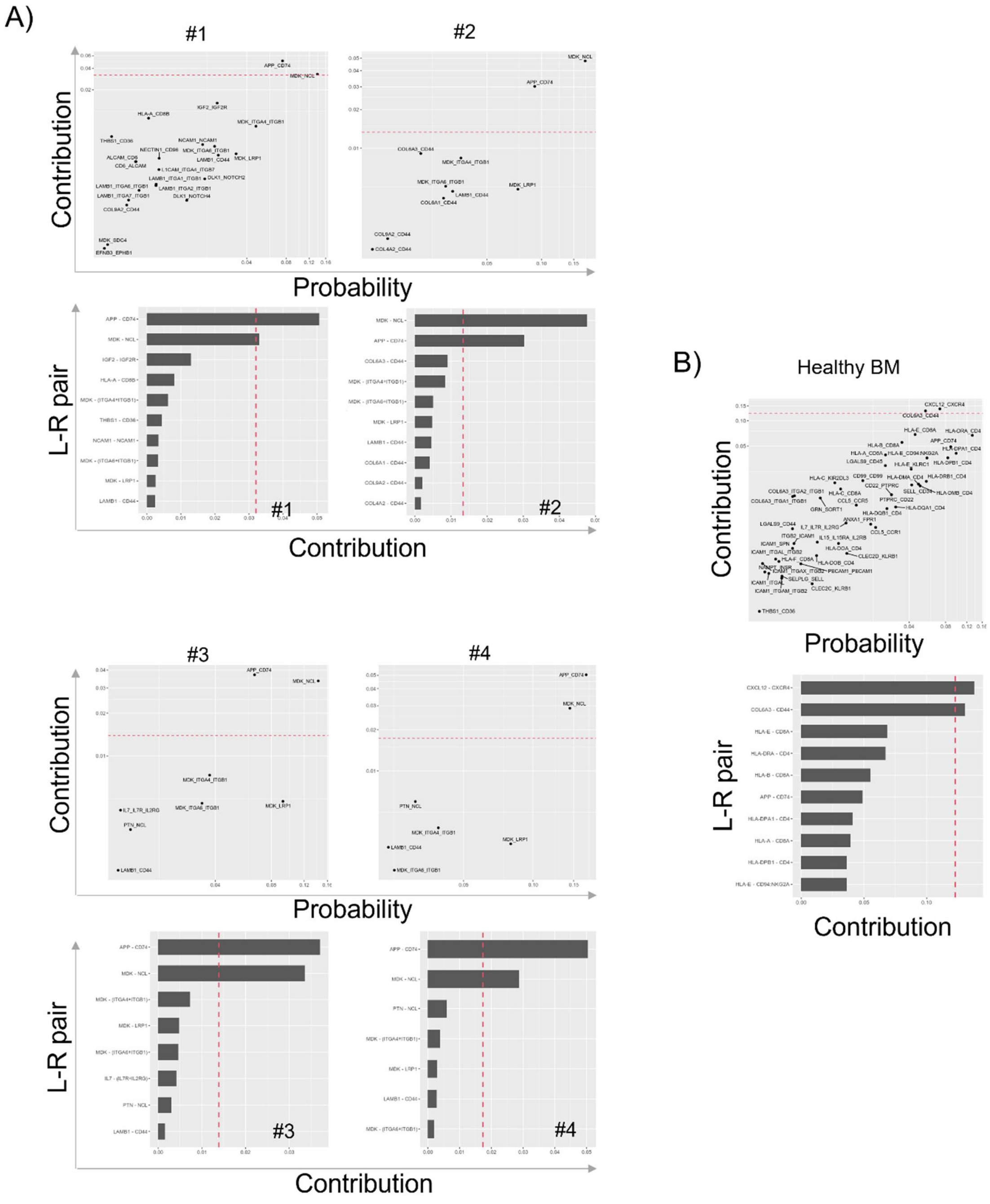

Figure S2. Inferred cell-cell communication by CellChat. A) Probability of ligand-receptor (L-R) pairs annotated for the contribution of the communication roadmap in the BM metastatic neuroblastoma samples analyzed (top panel). The top 10 L-R pairs ranked in each patient (#1, #2, #3, #4 - lower bar graph). B) The same analysis for healthy BM sample.

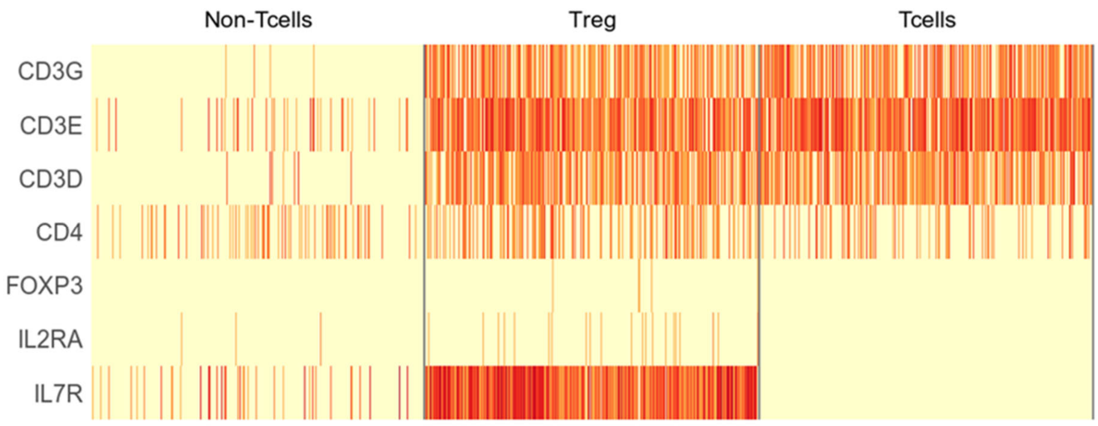

Figure S3. T^{reg} signature genes expression. The Heatmap reports the expression of the T^{reg} signature genes in CD3+-T cells (Tcells), T^{reg} cells and non-T cells sampled from the remaining cell populations described in the BM. Red color gradation depicts gene expression levels (red – high expression; yellow – low expression).

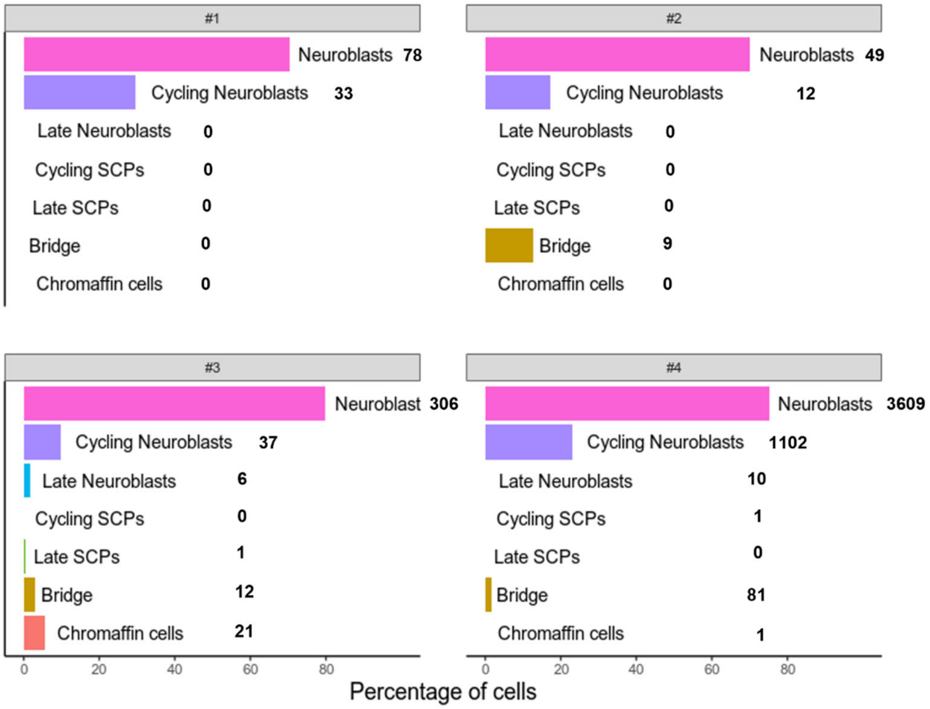

Figure S4. Proportions of neuroblastoma cells in metastatic BM. Percentage of various tumor cell populations in each of the four patients (#1, #2, #3, #4), according to their similarity to populations from human fetal adrenal medulla. The numbers (in bold) refer to the actual cell count determined in silico for each population.

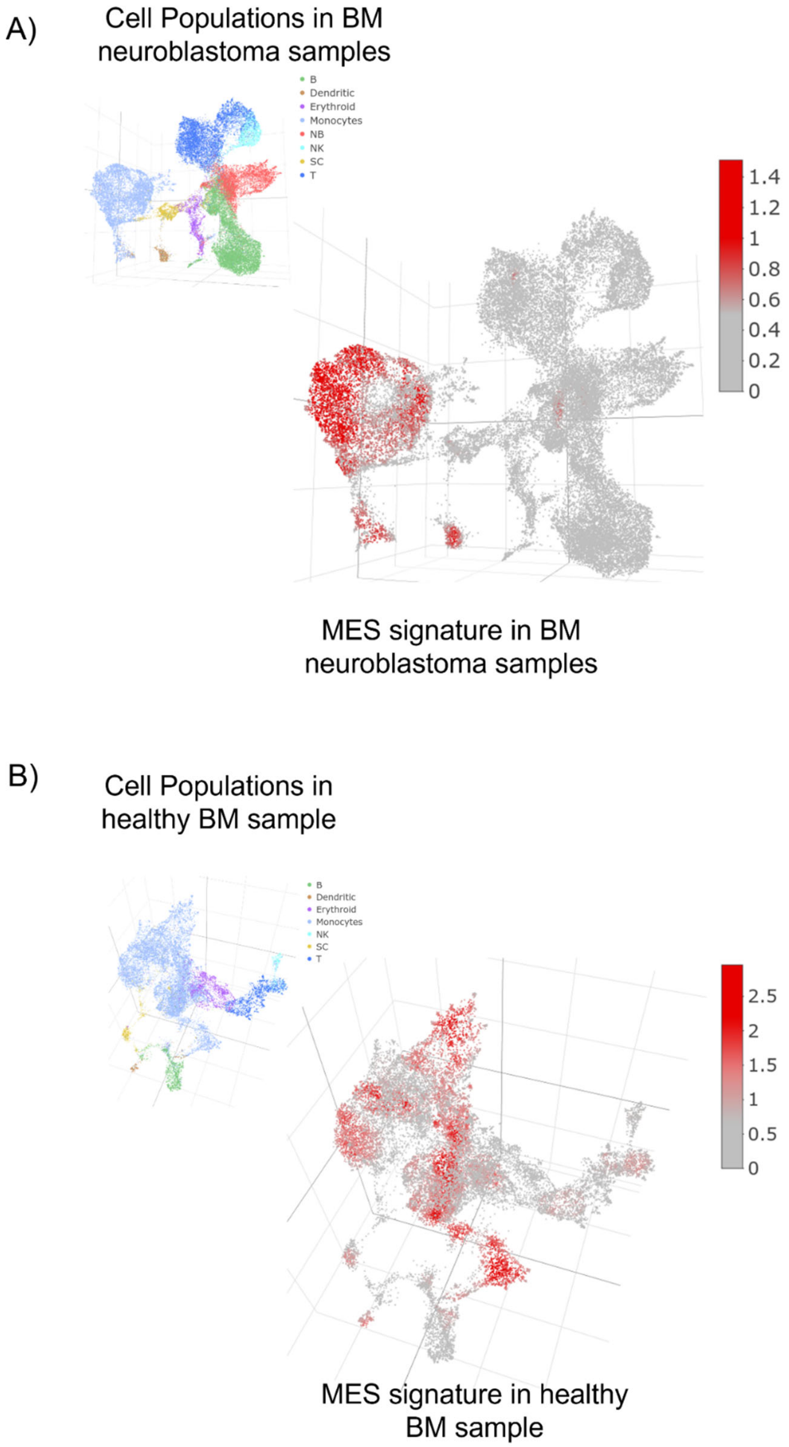

Figure S5. Mesenchymal (MES) signature distribution in the cell populations from: A) four BM metastatic neuroblastomas and B) heathy BM sample. Color gradation depicts MES

Z-score. Red indicates the maximum score. Different cell populations and their distribution are listed in the upper 3D UMAP included for each panel**.**

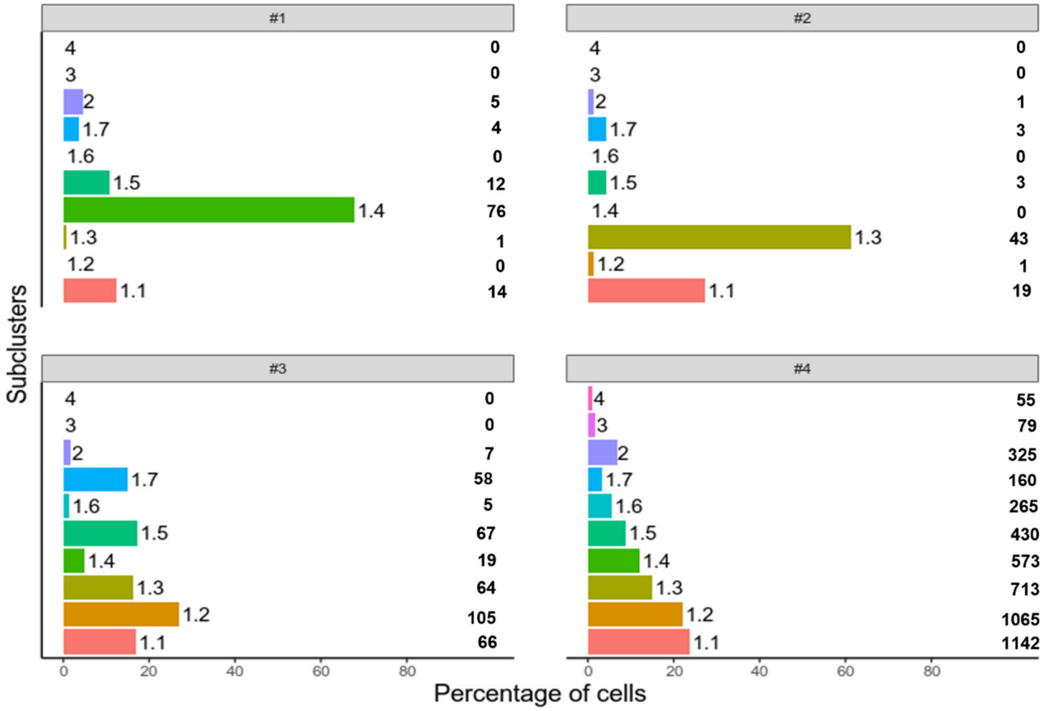

Figure S6. Proportions of neuroblastoma cells in metastatic BM. Percentage of various tumor cell subclusters (1.1; 1.2; 1.3; 1.4; 1.5; 1.6; 1.7; 2; 3 and 4) in each of the four patients (#1, #2, #3, #4). The numbers (in bold) refer to the actual cell count determined in silico for each subcluster.

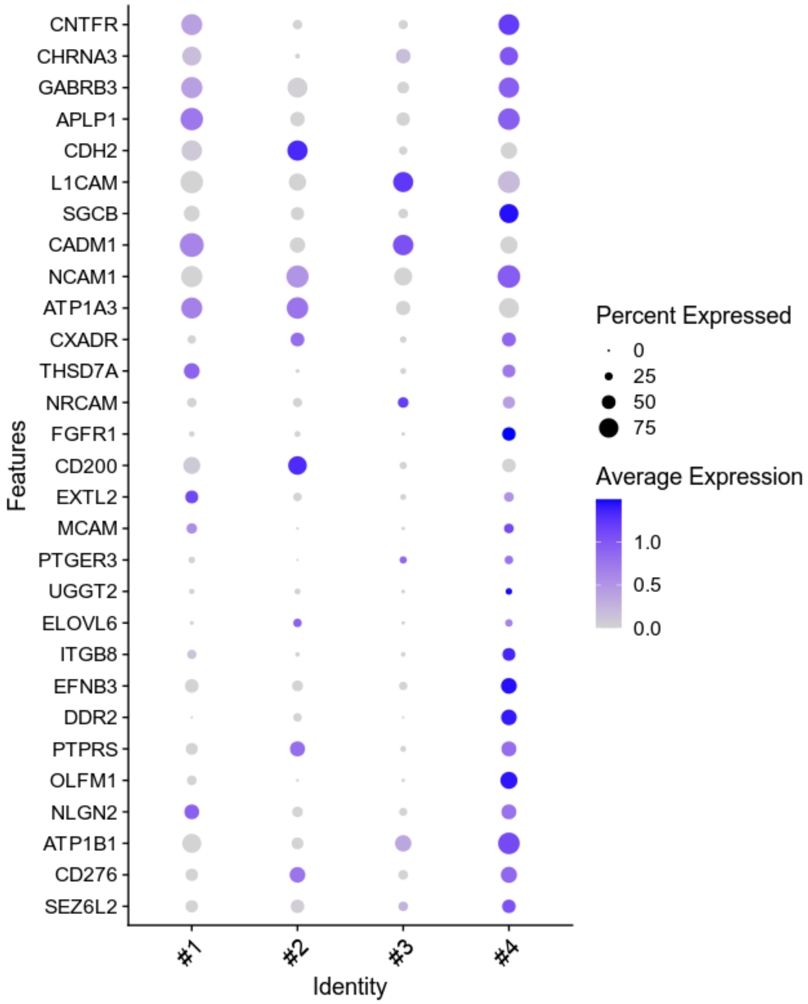

Figure S7. Expression patterns of TSA across analyzed specimens as dot plot. Dot size indicates percentage of cells expressing each gene, and color intensity represents average expression level.

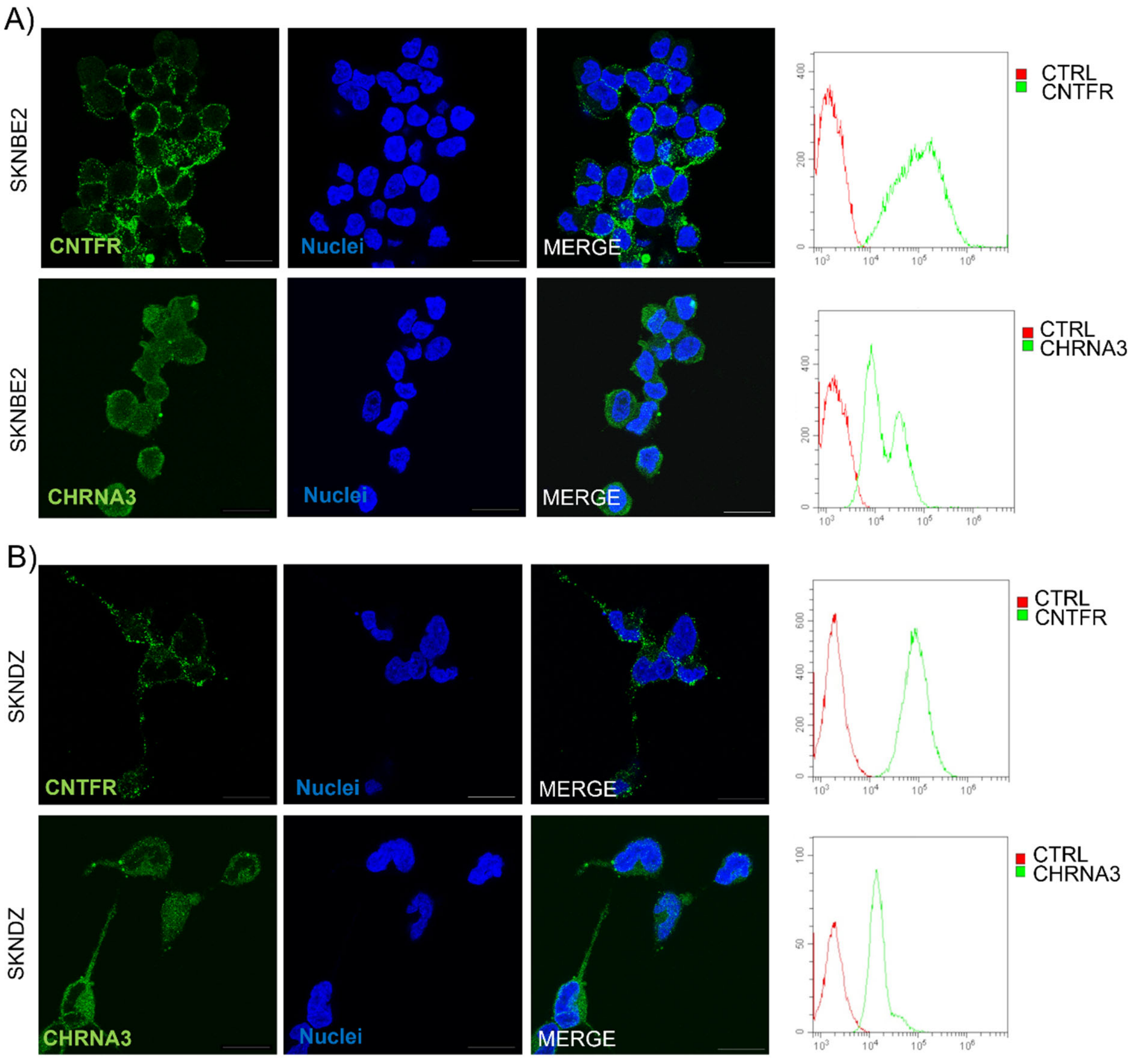

Figure S8. Expression of TSAs in different neuroblastoma cell lines. On the left – representative immunoreactivity for CNTFR and CHRNA3 TSAs validated on the membrane of neuroblastoma cells A) SKNBE2 and B) SKNDZ. Green – 488 coupled secondary antibodies. DAPI (blue) – nuclear staining. Scale bar: 20 μm. On the right – representative single measurement of the indicated antigens (TSAs – green; CTRL – red) using MFC is presented for each neuroblastoma cell line. Analysis is done using FlowJo. CTRL – control sample.

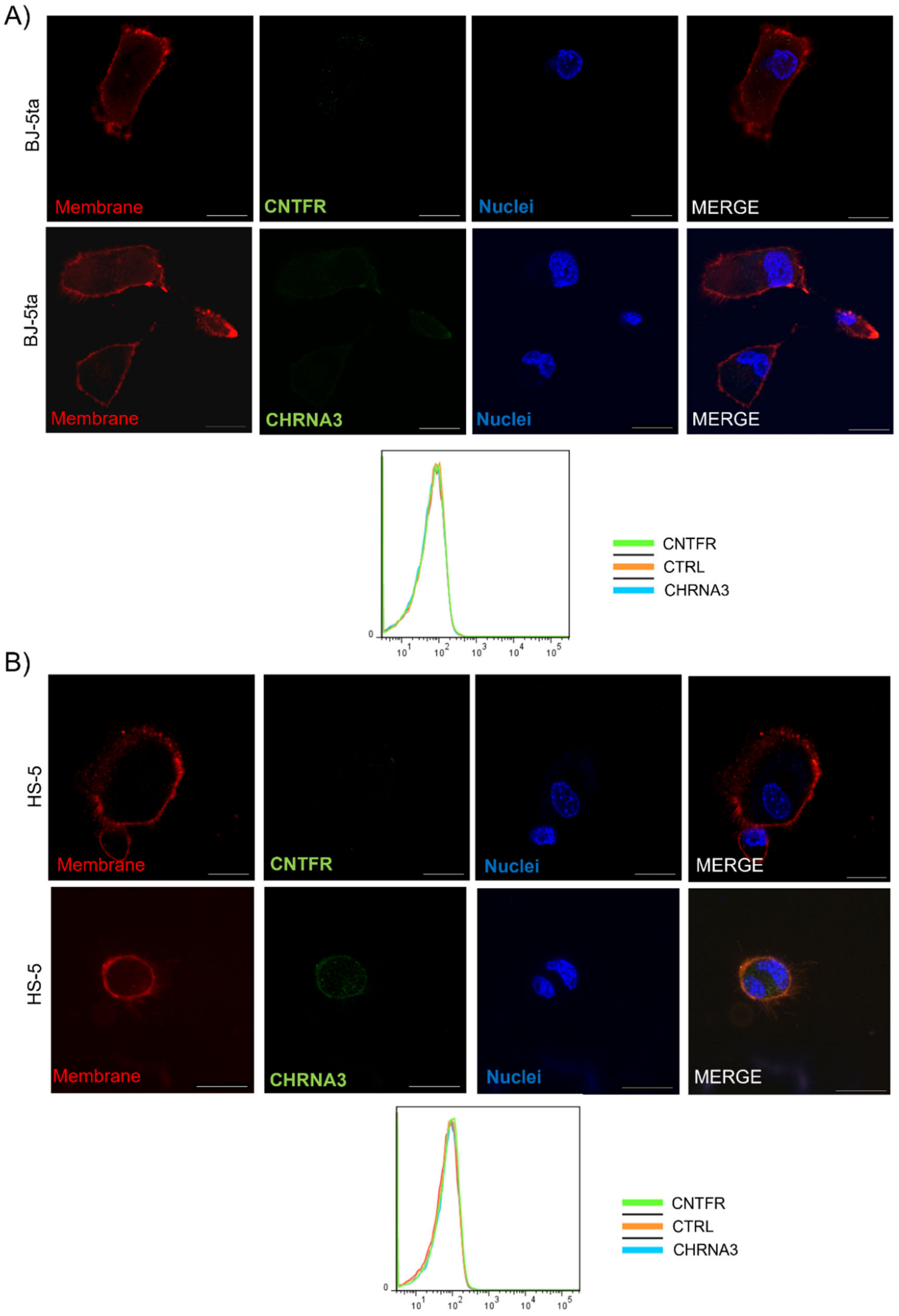

Figure S9. Expression of TSAs in non-malignant cells. Representative immunoreactivity of antibodies with each of the TSAs in A) BJ-5ta fibroblast and B) HS-5 mesenchymal cell line was checked with specific primary antibody coupled with 488 (green) secondary antibodies. Membrane is stained with MemBrite (red) – cell membrane specific dye. DAPI (blue) – nuclear

staining. Scale bar: 20 µm. Representative single measurement of the indicated antigens (CNTFR – green; CHRNA3 – blue; CTRL - orange) using flow cytometry is presented for each non-malignant cell line. Analysis is done using FlowJo. CTRL – control sample.

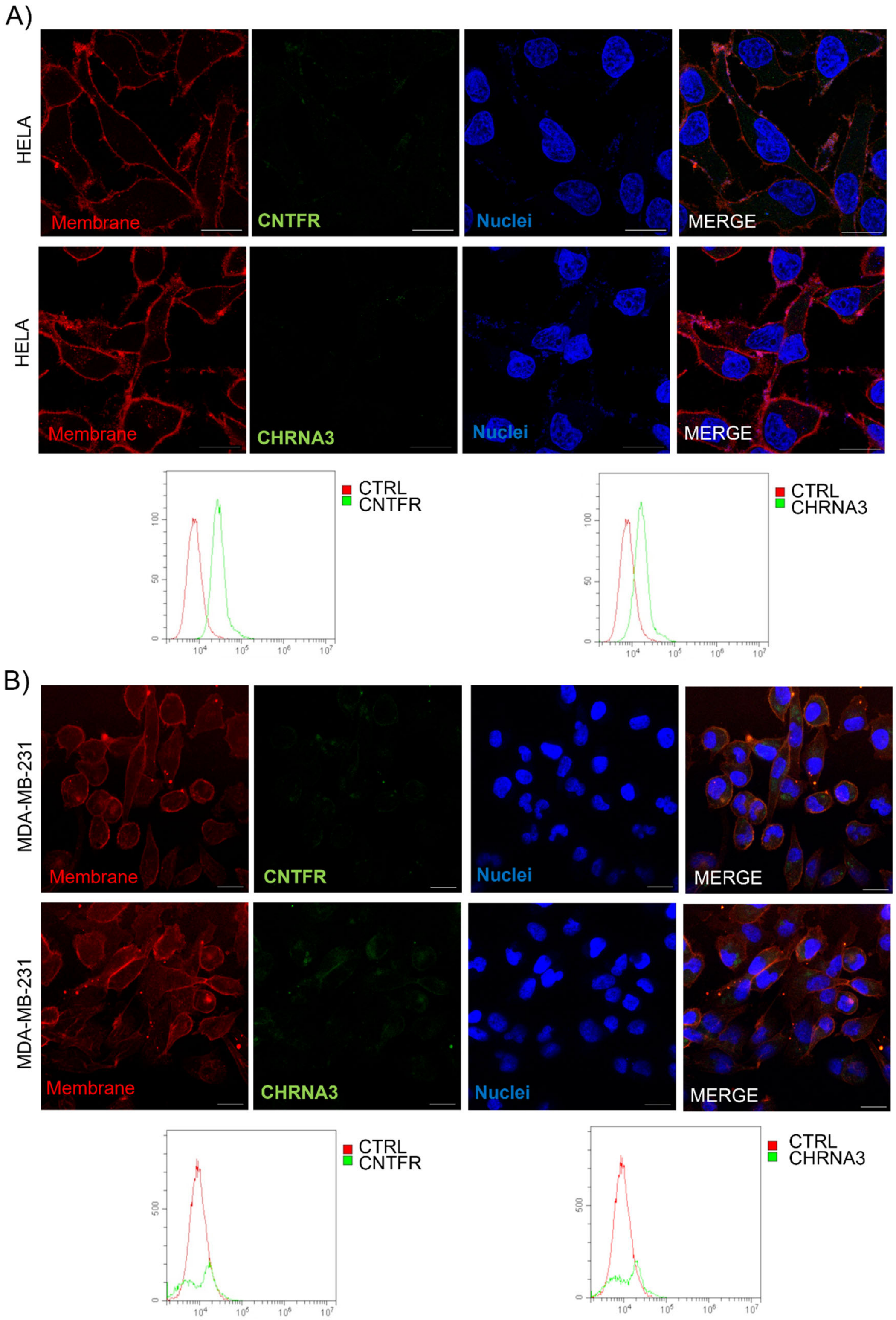

Figure S10. Expression of TSAs in non-neuroblastoma cancer cells. Representative immunoreactivity and flow cytometry readouts for the TSAs in A) HELA cervix cancer cell

line and B) MDA-MB-231 breast cancer cell line. Each primary antibody was coupled with an adequate 488 (green) secondary antibody. Membrane is stained with MemBrite (red) – cell membrane specific dye. DAPI (blue) – nuclear staining. Scale bar: 20 μm. CTRL – control sample.

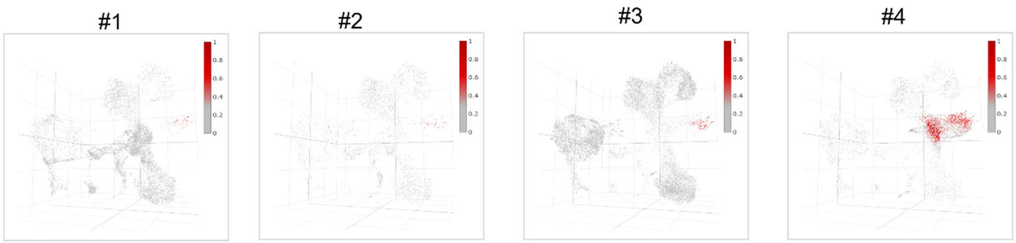

Figure S11. Assessment of 24 TSAs signature in the individual patient's BM specimens (numbers #1, #2, #3 and #4 indicate sample origin and correspond to the same samples described in Figure 2F).

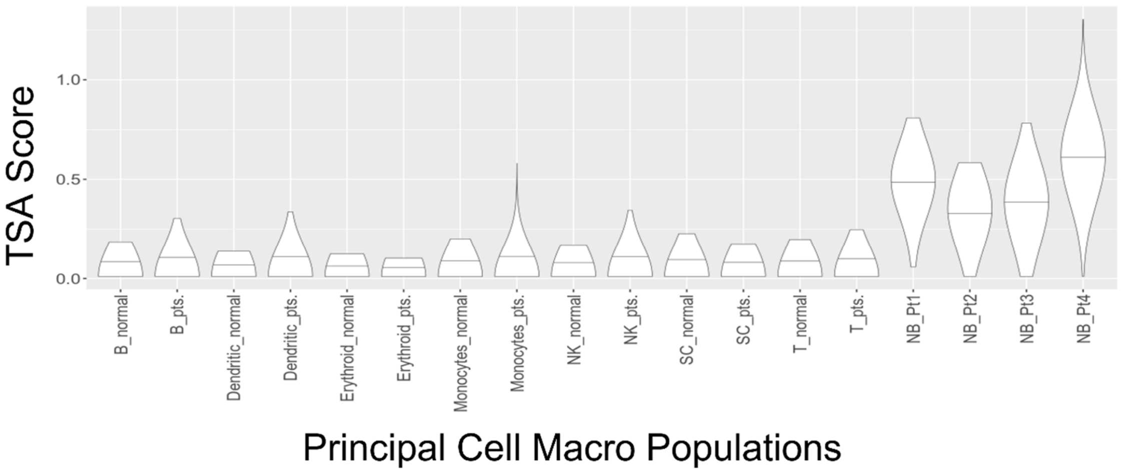

Figure S12. Assessment of the 24 TSAs signature (TSA score) in the principal cell macro populations of healthy BM specimen (normal) and patient derived samples (pts). Violin plots indicate a level of 24 TSA signature in principal cell macro-populations (corresponding to Figure S3A-B) obtained from patients with BM metastatic neuroblastoma (pts or pt1; pt2; pt3; pt4) and healthy BM (normal). NB – neuroblastoma; B – B cells; NK – NK cells; SC – stem cells; T – T cells. pts – refers to all four samples analyzed together.

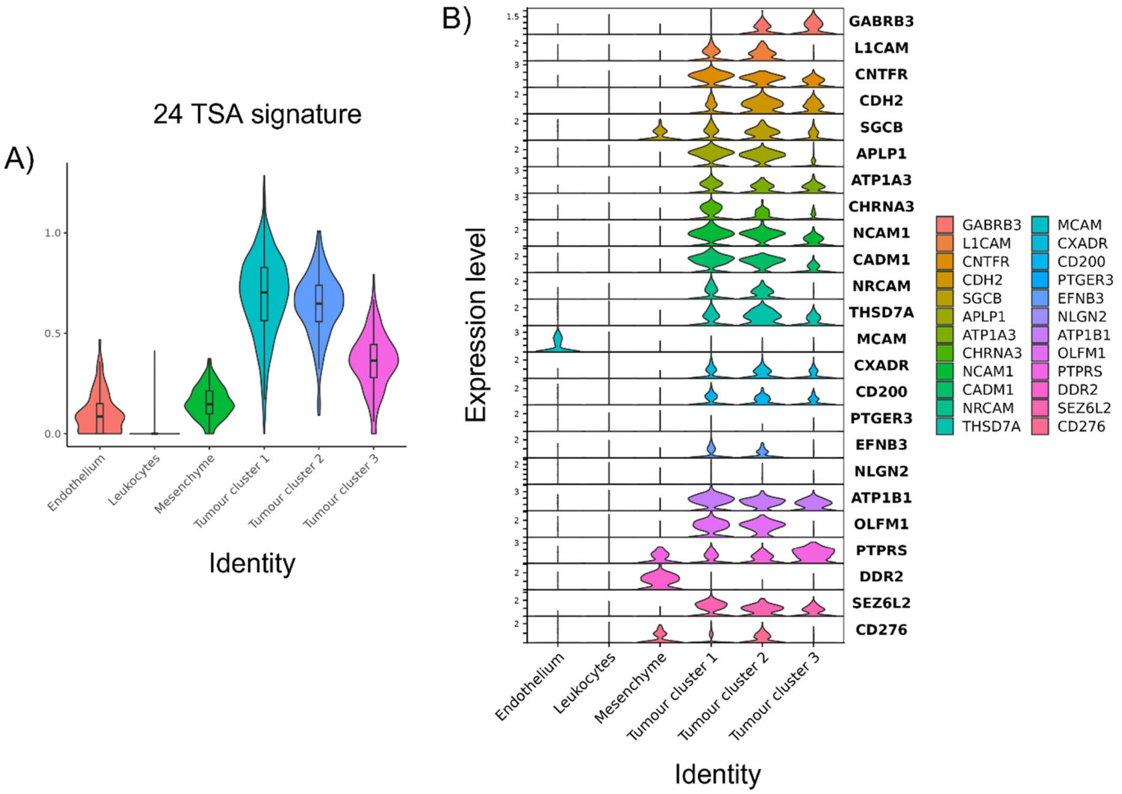

Figure S13. Assessment of 24 TSA signature A) as a signature (all together) or B) individually, in the publicly available scRNA-seq dataset obtained from diagnostic biopsies or neuroblastoma resections of primary tumors. Processed data is available at http://neuroblastomacellatlas.org.

Supplementary Tables

Table S1. Clinical features of patients.

Table S2. Cell lines and media.

Cell line	Cell type	Growth medium
SH-SY5Y	Neuroblastoma	DMEM with 20% FBS
SKNAS	Neuroblastoma	DMEM with 10% FBS and NEAA 1x (GIBCO; #11140-035)
SKNDZ	Neuroblastoma	DMEM with 10% FBS and NEAA 1x
SKNBE2	Neuroblastoma	RPMI with 10% FBS
HELA	Cervix carcinoma	RPMI with 10% FBS
MDA-MB-231	Breast carcinoma	RPMI with 10% FBS
HS-5	MSC	MSC Medium supplemented with Mesenchymal Stem Cell Growth Factors (ATCC; #PCS-500-030 and #PCS-500-040)
BJ5	Fibroblasts	DMEM:F12 (#AU-L0136-500; 1:1) with 2% FBS

Table S3. List of the antibodies used for immunostaining and MFC.

Antibody	Company	Cat. number
Mouse monoclonal AChRalpha3 (C-6)	Santa Cruz Biotechnology	Cat# sc-365479; RRID: AB_10847941
Mouse monoclonal CNTFRalpha (AN-B2)	Santa Cruz Biotechnology	Cat# sc-9993; RRID: AB_2083210
Mouse monoclonal FOXP3	Leica Biosystems	Cat# PA0263-U; RRID: AB_3698112
Mouse monoclonal CD3	Leica Biosystems	Cat# NCL-L-CD3-565; RRID: AB_563541
Goat anti-Mouse IgG (H+L) Cross-Adsorbed Secondary Antibody, Alexa Fluor™ 488	Thermo Fisher Scientific	Cat# A-11001; RRID: AB_2534069
CD4 FITC	BD Biosciences	Cat# 345768, RRID:AB_2868797
FITC Mouse Anti-Human CD94	BD Biosciences	Cat# 555888, RRID:AB_396200
CD57 FITC	BD Biosciences	Cat# 333169, RRID:AB_2728105
PE-Cy7 Mouse Anti-Human CD24	BD Biosciences	Cat# 561646, RRID:AB_10892826
APC Mouse Anti-Human CD133	BD Biosciences	Cat# 566597, RRID:AB_2744280
BV421 Mouse Anti-Human Disialoganglioside GD2	BD Biosciences	Cat# 564223, RRID:AB_2738679
BV510 Mouse Anti-Human CD9	BD Biosciences	Cat# 563640, RRID:AB_2738339

BV605 Mouse Anti-Human CD29	BD Biosciences	Cat# 743784, RRID:AB_2741752
BV711 Mouse Anti-Human CD56 (NCAM-1)	BD Biosciences	Cat# 740781, RRID:AB_2740444
BV786 Mouse Anti-Human CD45	BD Biosciences	Cat# 563716, RRID:AB_2716864
BV605 Mouse Anti-Human CD47	BD Biosciences	Cat# 563759, RRID:AB_2738408
BV510 Mouse Anti-Human CD49b	BD Biosciences	Cat# 743202, RRID:AB_2741340
BV421 Mouse Anti-Human CD279 (PD-1)	BD Biosciences	Cat# 565935, RRID:AB_11153482
APC-R700 Mouse Anti-Human LAG3 (CD223)	BD Biosciences	Cat# 565775, RRID:AB_2744329
AlexaFluor 647 Mouse Anti-Human TIM-3 (CD366)	BD Biosciences	Cat# 565559, RRID:AB_2744367
PE Mouse Anti-Human CD96	BD Biosciences	Cat# 562379, RRID:AB_2737612
BV510 Mouse Anti-Human CD73	BD Biosciences	Cat# 563198, RRID:AB_2738062
V500 Mouse Anti-Human HLA-DR	BD Biosciences	Cat# 561224, RRID:AB_10563765
V500 Mouse Anti-Human CD19	BD Biosciences	Cat# 561125, RRID:AB_10562391
CD3 APC-Cy7	BD Biosciences	Cat# 341110 RRID:AB_2868766
CD7 V450	BD Biosciences	Cat# 642921 RRID:AB_1645756
V450 Mouse Anti-Human CD19	BD Biosciences	Cat# 560353 RRID:AB_1645564
V450 Mouse Anti-Human CD38	BD Biosciences	Cat# 561378, RRID:AB_10689627
APC Mouse Anti-Human CD117	BD Biosciences	Cat# 341096, RRID:AB_400563
BB700 Mouse Anti-Human CD152	BD Biosciences	Cat# 566902, RRID:AB_2869943
PE-Cy5 Mouse Anti-Human CD33	BD Biosciences	Cat# 551377, RRID:AB_394173
PE Mouse Anti-Human CD44	BD Biosciences	Cat# 550989, RRID:AB_394000
PE-Cy7 Mouse Anti-Human CD25	BD Biosciences	Cat# 560920, RRID:AB_396847
PerCP-Cy5.5 Mouse Anti-Human CD16	BD Biosciences	Cat# 560717, RRID:AB_1727434
PE-Cy7 Mouse Anti-Human CD146	BD Biosciences	Cat# 562135, RRID:AB_10894593
PE Mouse Anti-Human CD151	BD Biosciences	Cat# 556057, RRID:AB_396327
PE Mouse Anti-Human CD200	BD Biosciences	Cat# 552475, RRID:AB_394398
PE Mouse Anti-Human CD83	BD Biosciences	Cat# 556855, RRID:AB_396526
PE Mouse Anti-Human CD8	BD Pharmingen	Cat# 345773, RRID:AB_2868801
CD24-PC5	Beckman Coulter	Cat# IM2645, RRID:AB_10053436
CD56-PC7	Beckman Coulter	Cat# A21692, RRID:AB_2892144
CD33-APC-Alexa Fluor 750	Beckman Coulter	Cat# C86905, Clone D3HL60.251
Anti-Hu CD99 PE	ExBio	Cat# 1P-658-T100, Clone 3B2/TA8

Table S4. Seurat 'FindAllMarkers' output of the top 5 (by avg_log2FC) DEGs per cluster used as a source for the heatmap of Fig.1C. "gene" is the gene symbol; "cluster" is the cluster in which genes are detected; "avg_log2FC" is the average Fold Change detected between the gene expression in the cluster and its expression in all the other clusters; "p_val" is p-value of the wilcoxon test; "p_val_adj" is the Bonferroni adjusted p_value; "pct.1" is the percentage of cells in the cluster where the gene is detected; "pct.2" is the percentage of cells on average in all the other clusters where the gene is detected.

Table S5. Immunophenotypic profile based on MFC for neuroblastoma cells infiltrating BM.

Antigen*	Pt 1	Pt 2	Pt 3	Pt 4	Pt 5	Pt 6	Pt 7	Pt 8	Pt 9	Pt 10	Rate	%
**CD56**	++	++	++	++	++	++	++	++	++	++	10/10	*100*
**CD117**	-	-	-	-	-	-	-	-	-	+	1/10	*10*
**CD99**	-	-	-	-	-	-	-	-	-	-	0/10	*0*
**CD45**	-	-	-	-	-	-	-	-	-	-	0/10	*0*
**CD24**	nd	++	++	++	++	++	++	++	++	++	9/9	*100*
**GD2**	nd	++	++	++	++	++	++	++	++	++	9/9	*100*
**CD133**	-	-	-	-	-	-	nd	+	++	-	2/9	*22*
**CD29**	-	-	-	++	-	++	-	-	nd	nd	2/8	*25*
**CD9**	-	-	+	-	++	++	++	++	+	-	6/10	*60*
**CD44**	nd	++	++	++	++	++	nd	+	nd	++	7/7	*100*
**CD94**	nd	-	-	++	-	-	-	-	nd	nd	1/7	*14*
**CD96**	nd	++	-	-	-	-	-	-	nd	nd	1/7	*14*
**CTLA4**	nd	-	-	++	-	-	+	-	nd	nd	2/7	*29*
**CD146**	nd	++	-	++	-	++	++	++	++	++	7/9	*78*
**TIM3**	nd	++	-	++	-	-	-	-	nd	nd	2/7	*29*
**LAG3**	nd	-	-	++	-	+	++	-	nd	nd	3/7	*43*
**PD1**	nd	-	-	-	-	-	-	-	nd	nd	0/7	*0*
**CD49b**	nd	+	-	-	-	-	-	-	nd	nd	1/7	*14*
**CD47**	nd	++	+	++	++	+	+	+	nd	nd	7/7	*100*
**CD73**	nd	-	-	+	-	-	-	++	-	-	2/9	*22*
**CD200**	nd	nd	nd	nd	++	++	++	++	nd	++	5/5	*100*
**CD57**	nd	nd	nd	nd	++	+	-	-	++	-	3/6	*50*
**CD151**	nd	nd	nd	nd	++	++	++	++	++	++	6/6	*100*
**CD34**	-	-	-	-	-	-	-	-	-	-	0/10	*0*
**CD90**	nd	nd	nd	nd	++	++	++	++	++	++	6/6	*100*
**CD38**	-	-	-	-	-	-	-	-	-	-	0/10	*0*
**CD83**	nd	nd	nd	nd	-	-	-	-	-	-	6/6	*100*
**HLADR**	nd	-	-	-	-	-	-	-	-	-	0/9	*0*

**CD7**	-	-	-	-	-	-	-	-	-	0/10	*0*
**CD3**	nd	-	-	-	-	-	-	-	-	0/9	*0*
**CD19**	nd	-	-	-	-	-	-	-	-	0/9	*0*
**CD25**	nd	-	-	-	-	-	-	-	-	0/9	*0*
**CD33**	-	-	-	-	-	-	-	-	-	0/10	*0*

*Antigen expression was rated into positive strong (++), positive weak (+) and negative (-), according to the fluorescence shift of neuroblastoma cell population compared to appropriate internal controls. **nd – non determined.**

Table S6. 'FindAllMarkers' output of the TSA genes. "Gene" is the gene symbol; "cluster" is the cluster in which genes are detected; "avg_log2FC" is the average Fold Change detected between the gene expression in the cluster and its expression in all the other clusters; "p_val" is p-value of the wilcoxon test; "p_val_adj" is the Bonferroni adjusted p_value; "pct.1" is the percentage of cells in the cluster where the gene is detected; "pct.2" is the percentage of cells on average in all the other clusters where the gene is detected. "All.NB" indicates that the gene is a differentially expressed gene (DEG) for all neuroblastoma (NB) clusters compared to normal. pm_score from 3-5 refers to the higher possible score (among values from 0-5) for a membrane location of the selected TSAs.

Table S7. Differentially expressed genes (DEGs) detected in bulk RNA-seq. "Gene" is the gene symbol; "logFC" is the base 2 logarithm of the fold change in expression between the two conditions analyzed (DTC&TU vs MNC); "AveExpr" is the average expression level of the gene across all samples. "P.Value" is the p-value calculated from a moderated t-statistic based on a linear model fitted to the gene expression data using the limma package; "adj.P.Val" is the p-value adjusted for multiple testing using the Benjamini-Hochberg method. pm_score from 3-5 refers to the highest possible score for a membrane localization of the selected TSAs.
